# Information Thermodynamics of Cytosine DNA Methylation

**DOI:** 10.1371/journal.pone.0150427

**Published:** 2016-03-10

**Authors:** Robersy Sanchez, Sally A. Mackenzie

**Affiliations:** Department of Agronomy and Horticulture, University of Nebraska-Lincoln, Lincoln, Nebraska, United States of America; CNRS UMR7275, FRANCE

## Abstract

Cytosine DNA methylation (CDM) is a stable epigenetic modification to the genome and a widespread regulatory process in living organisms that involves multicomponent molecular machines. Genome-wide cytosine methylation patterning participates in the epigenetic reprogramming of a cell, suggesting that the biological information contained within methylation positions may be amenable to decoding. Adaptation to a new cellular or organismal environment also implies the potential for genome-wide redistribution of CDM changes that will ensure the stability of DNA molecules. This raises the question of whether or not we would be able to sort out the regulatory methylation signals from the CDM background (“noise”) induced by thermal fluctuations. Here, we propose a novel statistical and information thermodynamic description of the CDM changes to address the last question. The physical basis of our statistical mechanical model was evaluated in two respects: 1) the adherence to Landauer’s principle, according to which molecular machines must dissipate a minimum energy *ε* = *k*_*B*_*T ln*2 at each logic operation, where *k*_*B*_ is the Boltzmann constant, and *T* is the absolute temperature and 2) whether or not the binary stretch of methylation marks on the DNA molecule comprise a language of sorts, properly constrained by thermodynamic principles. The study was performed for genome-wide methylation data from 152 ecotypes and 40 trans-generational variations of *Arabidopsis thaliana* and 93 human tissues. The DNA persistence length, a basic mechanical property altered by CDM, was estimated with values from 39 to 66.9 nm. Classical methylome analysis can be retrieved by applying information thermodynamic modelling, which is able to discriminate signal from noise. Our finding suggests that the CDM signal comprises a language scheme properly constrained by molecular thermodynamic principles, which is part of an epigenomic communication system that obeys the same thermodynamic rules as do current human communication systems.

## Introduction

Plant and animal phenotypes respond to environmental changes, an adaptive capacity that is, at least in part, trans-generational. Genetic and epigenetic factors are involved in a phenotypic range of this response. The genome-wide cytosine DNA methylation patterning that participates in the epigenetic response of cells to environmental variation is controlled by a complex network of genes. Cytosine DNA methylation (CDM) results from the addition of methyl groups to cytosine C5 residues, and the configuration of methylation within a genome provides trans-generational epigenetic information. The biochemical reaction is catalyzed by methyltransferases recruited into complex multicomponent molecular machines [1]. The reverse process of methyl group removal is catalyzed by demethylases [2]. These epigenetic modifications can influence the transcriptional activity of the corresponding genes, or maintain genome integrity by repressing transposable elements and affecting long-term gene silencing mechanisms [1,3].

Analysis of the biophysical mechanisms associated with cytosine methylation, and how these mechanisms can potentially explain the functional impact of cytosine methylation, has been described [4,5]. CDM alters the mechanical properties of a DNA molecule, particularly its flexibility [6–10]. Experimental evidence to date indicates that CDM plays an important role in preserving the stability of DNA [10–14]. As a consequence, we assume that adaptation of an individual to a new environment induces regulatory methylation responses (biological signal) that would likewise ensure DNA stability.

At a molecular level, the uncertainty of methylation status at each single cytosine site primarily derives from the omnipresent thermal fluctuations [5,6,9,10] that, in addition, impact the kinetics of biomolecular systems [6,15–18]. Thus, spontaneous methylation variation can be observed across generations, which may also affect gene expression [19,20].

Uncertainty of methylation status would be manifest even in a dataset generated from an “ideal zero error experiment” with perfectly synchronized cell samples. At a tissue level, this uncertainty derives from the several biological processes (e.g., differentiation, reprogramming, disease transformations) that involve cell transitions through distinct states [21]. In natural environments, cells from the same tissue are not necessarily in the same state and, therefore, corresponding cytosine sites differ in methylation status. Consequently, overall organismal response is conveyed as a statistical outcome, requiring an ability to distinguish regulatory methylation signals from the CDM statistical background (“noise”) induced by thermal fluctuations. Solving this problem transcends current state of the art in methylation analysis, which relies predominantly on *ad hoc* concepts of differentially methylated positions (DMPs) and differentially methylated regions (DMRs) defined by statistical tests that ignore the biochemical and biophysical (thermodynamic) nature of the genome-wide methylation process.

Based on statistical biophysics subjacent to CDM, we propose a novel statistical mechanical approach to describe the information thermodynamics of CDM changes and to confront the problem of methylation regulatory signal detection. We assume that if a significant proportion of the methylation changes induced by thermal fluctuation serve to stabilize the DNA molecule, then these changes will conform to statistical mechanical principles. In particular, the minimal energy dissipated to process the information associated with these methylation changes should follow statistical mechanical probability distributions. This energy is determined by Landauer’s principle, according to which, a molecular machine must dissipate a minimum energy of *ε* = *k*_*B*_*T ln*2 (about 3 × 10^−21^ Joules at room temperature) at each step in the genetic logic operations including proofreading [22,23]. This is the expected minimal energy dissipation that a molecular machine must spend to produce a change in one bit of information.

The physical foundation of our statistical mechanical model was evaluated by the estimation of a basic molecular property of DNA molecules, the DNA persistence length *L*_*p*_. The value of *L*_*p*_ indicates the maximum length of a polymer before thermal motion forces it to fluctuate wildly. The consensus value from estimations of DNA persistence length ${\hat{L}}_{p}$ is about 50 nm (~150 bp) [24,25], although estimated values of ${\hat{L}}_{p}$ can vary depending on ionic strength [26,27]. Evidence suggests that methylated ds-DNA has a substantially higher persistence length than non-methylated DNA [10], reaching about 92.5 nm when 9% of the total DNA is methylated. This effect increases rigidity of the DNA molecule and increases nucleosome compaction and rigidity [10,28].

Here we present theoretical and experimental validation for the statistical mechanical model, as well as definitions involving its preliminary application to methylation analysis. Results suggesting the existence of a methylation language consistent with our statistical mechanical modeling are also described. For simplification, all equations used to derive the presented information are provided in the Materials and Methods section.

## Results and Discussion

The absolute amount of information *I*_*R*_ processed by the methylation machinery in the genomic region *R* was estimated from Arabidopsis and human methylomes (Eq 3). Under Landauer’s principle, the minimum energy dissipated to process the information *I*_*R*_ can be expressed by the equation: *E*_*R*_ = *I*_*R*_*k*_*B*_*T ln*2 (Eq 4). Based on simple physical assumptions, the probability density function (PDF) for the energies *E*_*R*_ was approached by a Generalized Gamma distribution (GG, Eq 7). This probability distribution accounts for an informational statistical thermodynamics description of methylation changes induced by thermal fluctuations, which are presumed to stabilize the DNA molecule. These methylation changes represent “methylation background noise” with respect to the signal created by the methylation regulatory machinery. However, since methylation changes alter the mechanical properties of the DNA molecule [6], any methylation signal created by the methylation regulatory machinery also implies a redistribution of CDM changes for DNA stability. So, the knowledge of the probability distribution followed by the methylation background noise provides an analytical way to discriminate it from the biological signal [29–31].

Because GG distribution comprises a family of distributions, the best physical description of methylation background noise could be found in any member of the family. In this case, Weibull distribution, a member of the GG distribution family, was also identified in two ways that are presented in S1 Appendix. The Weibull distribution was derived under the assumption that the dissipation of the energies *E*_*R*_ follows a binomial process or a Poisson process, which in turn is derived as the limiting case of the former. To define a binomial (or a Poisson) process, the numbers of CDM changes induced by thermal fluctuations in non-overlapping genomic regions must be independent for all genomic regions. If the CDM changes induced by thermal fluctuations are consistent with a binomial (Poisson) process, then we can distinguish these CDM changes from those originated from the methylation regulatory machinery, which are not independent for all genomic regions.

Robust estimations of GG and Weibull cumulative distribution functions (CDFs, Eqs 12 and 14) were obtained for non-overlapping regions of 2000 to 5000 bp (S1 and S2 Tables and S1 Fig). Also, in a section (below) on the binary language of cytosine DNA methylation, we show that the architecture of small clusters (“words”) of CDM not only are characterized based on maximum entropy and least effort principles, but also fits the statistical mechanics given by Weibull distribution on statistical and physical basis.

In the next section we discussed statistical and physical evidence retrieved from the experimental data that support the application of Weibull distribution to discriminate the biological signal from methylation background noise.

### Statistical mechanical basis of the information thermodynamic model

**Fig 1:**
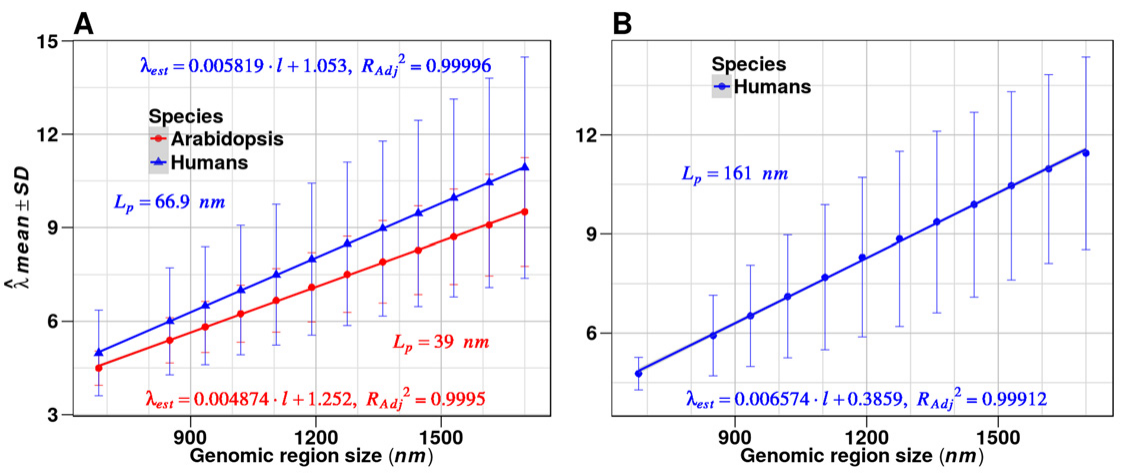
Statistical mechanical basis of the information thermodynamic model. (A) Analysis based on Weibull distribution. (B) Analysis based on GG distribution. Under Landauer’s principle statistical mechanical probability distributions were derived for the absolute amount of information *I*_*R*_ processed by the methylation machinery in a genomic region *R* (Eqs 12 and 14, Material and Methods). For each partition of the methylome into genomic regions, nonlinear fits were performed to estimate the scaling parameter $\hat{\lambda }\left(l\right)$. Next, regression functions were obtained from the regression analyses $\bar{\hat{\lambda }}\left(l\right)$ versus *l*, as predicted by Eq 18 (19). The estimations of the DNA persistence length ${\hat{L}}_{p}$ were based on Eq 20.

Although for each nonlinear fit we estimated Akaike and Bayesian information criteria [32,33], the final model selection also relied on whether or not the value of the scaling parameter $\hat{\lambda }\left(l\right)$, numerically estimated, was meaningful from a physical perspective. The scaling parameter *λ*(*l*) (from Eqs 12 and 14) conveys the contribution of all degrees of freedom to the average energy per molecule. For both Arabidopsis and human methylome datasets, the best fit was obtained by Weibull distribution (Fig 1).

In the case of Arabidopsis, the numerical algorithm used in the nonlinear fit of GG distribution yielded extremely low values of $\hat{\lambda }\left(l\right)$, approaching zero, which is meaningless from a physical standpoint. For the case of human methylomes, high and low extremes for $\hat{\lambda }\left(l\right)$ values were also observed in several samples. The introduction of constraints in the numerical algorithm for the interval of possible values for $\hat{\lambda }\left(l\right)$ improved the estimations, but it did not solve the fitting issue for all samples (see below).

As presented in Fig 1, fitting the human methylome data to Weibull and GG distribution permitted estimation of the DNA persistence length *L*_*p*_. In the current case, the ${\hat{L}}_{p}$ estimations for Arabidopsis and human methylomes obtained through Eq 20 and the regression analyses, $\bar{\hat{\lambda }}\left(l\right)$ versus region length *l* (Fig 1A, based on Weibull distribution), yielded values consistent with those reported in the literature [24–27]. Estimation based on GG distribution overestimated the values of *L*_*p*_ (Fig 1B), perhaps consistent with the finding that GG distribution improperly fit several samples from the human methylome dataset. However, this result doesn’t imply a general rejection of GG distribution, since different samples and genomic partitioning could fit the GG statistical model or another member of the GG distribution family as well.

Although the experimental data used for this analysis were from different species and obtained by different research groups, the results obtained for Weibull distribution remained consistent. Thus, under Landauer’s principle, Eqs 12 and 14 yield a statistical mechanical description of the information thermodynamics of CDM changes that occur in genomic regions.

### Differentially informative methylated positions

The knowledge of the statistical mechanical CDFs followed by the methylation background noise provides the tools for a robust estimation of differentially methylated position (DMP). A DMP is a single genomic position for which a significant statistical difference between the methylation levels from two different samples or two groups of samples is detected by the application of a suitable statistical test. Several statistical tests have been proposed to assess the detection of DMPs, including Fisher’s exact test, binomial test, logistic regression and beta binomial regression [34].

Two main sources of bias are present when DMPs are estimated by considering only the experimental data and the statistical test to evaluate the differences between samples. The first source of bias is introduced by ignoring the biophysical nature of the methylation process. In consequence, the classical methylation analysis is not able to sort out the regulatory methylation signals from the methylation background noise. Any statistical test to estimate DMPs must consider the statistical thermodynamics subjacent and inherent to the methylation process [4–7]. A second source of bias is introduced when a high number of multiple comparisons is performed. Adjustment of *p*-values is required for multiple comparisons and, in consequence, a number of potential DMPs can be rejected. Several algorithms/strategies have been proposed to confront this issue [34]. However, the application of these approaches to detect DMPs can lead to subjective results. At a tissue level, DMPs are the result of statistical-biophysical events that depend on the cells’ capacities to perform physical work. Thus, a DMP represents an objective difference that does not depend on the statistical test or the algorithm used to detect it, but rather, the magnitude of energy dissipated to produce it.

A formal definition of DMP can be derived based on the energy dissipated to produce a divergence between methylation levels. Eq 3 permits not only estimation of the uncertainty variation at a single cytosine position, but also the divergence between methylation levels. Results indicate that three other *information divergence* measures also express the divergence between methylation levels consistent with the theory developed for *I*_*R*_: Total-variation (*TV*, Eq 25), Kullback–Leibler (*KL*, Eq 26) and Hellinger (*H*^*D*^, Eq 27).

A formal definition of DMP inspired by the signal detection theory (STD) can be proposed [29–31]. Let $P\left(E_{k}^{D}\leq E_{k}^{D_{0}}\right)$ be the probability that energy $E_{k}^{D}$, dissipated to create an observed divergence *D*_0_ between the methylation levels from two different samples at a given genomic position *k*, can be lesser than or equal to the amount of energy $E_{k}^{D_{0}}$. Then, a single genomic position *k* shall be called a DMP at a level of significance *α* if, and only if, the probability $P\left(E_{k}^{D}>E_{k}^{D_{0}}\right)=1-P\left(E_{k}^{D}\leq E_{k}^{D_{0}}\right)$ to observe a methylation change with energy dissipation higher than $E_{k}^{D_{0}}$ is lesser than *α*. With this definition we want to emphasize the statistical-biophysical nature of DMPs at tissue or organ levels.

The above definition is intuitive from a biophysical perspective. Since Eqs 12 and 14 were derived on physical basis, these CDFs do not explain the methylation changes originated by the methylation regulatory machinery. Hence, a biological signal created by the regulatory methylation machinery can also be originated by statistical mechanic processes affecting the DNA molecule with probability $P\left(E_{k}^{D}>E_{k}^{D_{0}}\right)=1-P\left(E_{k}^{D}\leq E_{k}^{D_{0}}\right)$. According to the SDT, $P\left(E_{k}^{D}>E_{k}^{D_{0}}\right)$ is the probability of false positive, i.e., the probability to accept a cytosine methylation change as a DMP created by the methylation regulatory machinery when in fact it was created to stabilize the DNA molecule [31]. SDT provides the means to establish a threshold *α* to minimize the risk and to increase the sensitivity of DMP detection.

In practice, probabilities $P\left(E_{k}^{D}\leq E_{k}^{D_{0}}\right)$ can be approached by giving specific values to the divergence *D*_*R*_ in Eq 29 (for *R* = *k*, for brevity, any reference to Eq 29 will take into account its particular cases as well). A conservative approach can be, for example, $D_{R}=H_{R}^{D}$. That is, $P\left(E_{k}^{D}\leq E_{k}^{D_{0}}\right)≅P\left(H_{k}^{D}\leq {\hat{H}}_{k}^{D_{0}}\right)$ provided that $H_{k}^{D}$ is proportional to $E_{k}^{D}$, where the Hellinger divergence ${\hat{H}}_{k}^{D_{0}}$ is estimated from the experimental data according Eq 27 and the probabilities $P\left(H_{k}^{D}\leq {\hat{H}}_{k}^{D_{0}}\right)$ are estimated by means of Eq 29 for *R* = *k* and substituting $D_{k}={\hat{H}}_{k}^{D_{0}}$.

**Fig 2:**
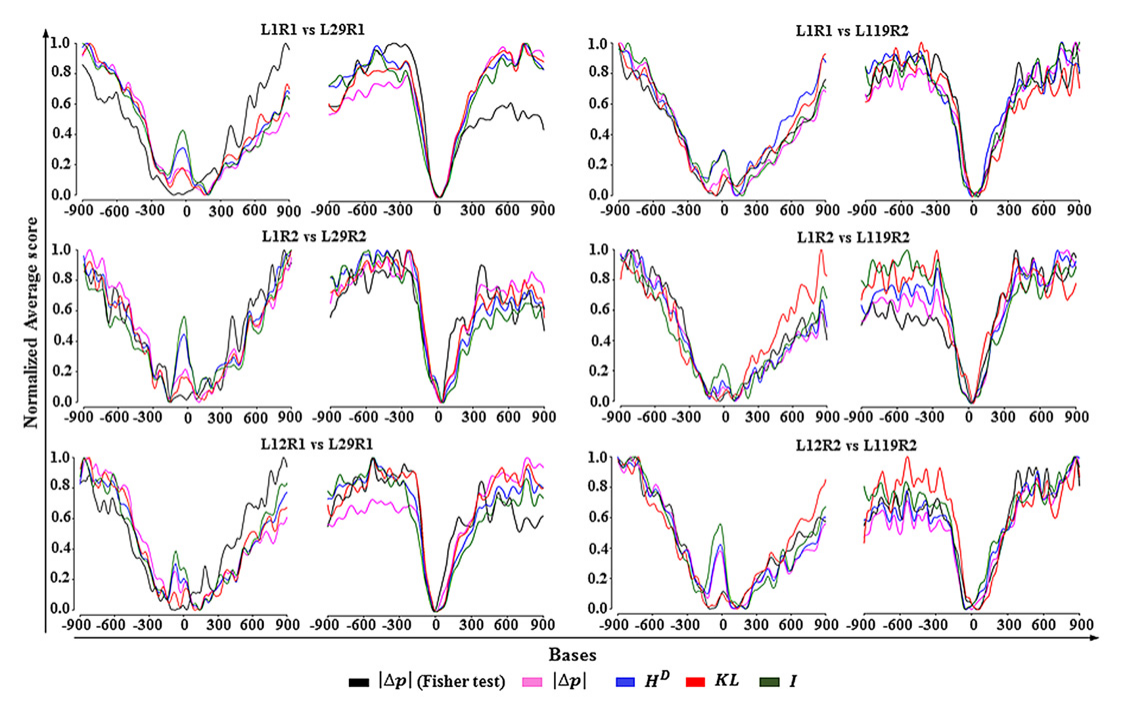
Density of DIMPs around transcription start and end sites. In each graphic the bases at position zero denote the centers of the 3’ (left) and 5’ (right) untranslated regions. The DMPs are estimates for the 30^th^ generation lines: L29 and L119 (replicates 1 and 2) in respect to 3^rd^ generation lines: L1 and L12 from reference [19]. Fisher exact test and the corresponding particular cases of Eq 30 (*D*_*R*_ = *D*_*k*_) for information (Eq 3, *D*_*k*_ = *I*_*k*_), Total variation (Eq 25, *D*_*k*_ = *TV*_*k*_), Kullback–Leibler (Eq 26, *D*_*k*_ = *KL*_*k*_) and Hellinger ($D_{k}=H_{k}^{D}$, Eq 27) divergences were used in the estimation of DMPs without distinction between methylation contexts. The density of DIMPs based on the absolute difference of methylation levels (which is equal to *TV*) was estimated based on Fisher exact test (*TV* (Fisher test)) and on the CDF for *TV* according to Eq 30 (*D*_*k*_ = *TV*_*k*_). DMPs estimated by the “classical” methods can be overestimated or underestimated. Any method to estimate DMPs must take into consideration the statistical-biophysical nature of the methylation process at tissue or organ levels. Every sample follow an independent ontogenetic development and the action of the omnipresent thermal fluctuations on cells and tissues leads to different methylation profiles.

The analysis on which *information divergence* can give the least biased estimation of $P\left(E_{k}^{D}\leq E_{k}^{D_{0}}\right)$ is a subject for further studies. Nevertheless, a DMP detected based on an information divergence measure shall be termed a differentially informative methylated position (DIMP). Densities of DIMPs detected using the cumulative distribution function of *TV*, *KL* and *H*^*D*^ in genomic regions close to the start and end sites of genes are similar to density profiles originating by Fisher’s exact test (Fig 2). Notice that the densities presented in Fig 2 only express general statistical tendencies and that, at single cytosine positions, Fisher’s exact test will coincide with statistical mechanical models only for extreme methylation changes.

The correlations between DMP density profiles are shown in Table 1. Since DMPs are the result of statistical-biophysical events, this is an expected outcome. However, this result does not mean that DMPs detected by Fisher’s exact test coincide at every cytosine position with those detected by the information divergences.

**Table 1:** Correlation between the densities of DMPs around transcription start and end sites.

L1R1 *vs* L29R1^a^						L1R1 *vs* L119R2 ^a^				
	*TV*_F_ ^b^	*TV*	*H*^*D*^	*KL*	*I*	*TV*_F_	*TV*	*H*^*D*^	*KL*	*I*
*TV*_F_	1.00	0.68	0.74	0.79	0.68	1.00	0.95	0.92	0.95	0.91
*TV*	0.57	1.00	0.97	0.97	0.95	0.90	1.00	0.93	0.94	0.97
*H*^*D*^	0.75	0.92	1.00	0.97	0.98	0.86	0.91	1.00	0.94	0.93
*KL*	0.70	0.96	0.95	1.00	0.94	0.89	0.89	0.88	1.00	0.90
*I*	0.68	0.88	0.98	0.89	1.00	0.84	0.92	0.94	0.83	1.00
L1R2 *vs* L29R2						L1R2 *vs* L119R2				
*TV*_F_	1.00	0.87	0.85	0.89	0.79	1.00	0.96	0.94	0.83	0.89
*TV*	0.76	1.00	0.94	0.97	0.87	0.85	1.00	0.99	0.82	0.94
*H*^*D*^	0.75	0.93	1.00	0.95	0.97	0.81	0.97	1.00	0.83	0.96
*KL*	0.78	0.96	0.95	1.00	0.88	0.69	0.75	0.80	1.00	0.83
*I*	0.70	0.88	0.98	0.90	1.00	0.62	0.83	0.90	0.77	1.00
L12R1 *vs* L29R1						L12R2 *vs* L119R2				
*TV*_F_	1.00	0.72	0.81	0.82	0.79	1.00	0.92	0.91	0.93	0.85
*TV*	0.59	1.00	0.96	0.97	0.91	0.92	1.00	0.99	0.86	0.94
*H*^*D*^	0.78	0.84	1.00	0.97	0.98	0.91	0.98	1.00	0.86	0.96
*KL*	0.77	0.89	0.97	1.00	0.92	0.84	0.79	0.81	1.00	0.81
*I*	0.75	0.75	0.98	0.86	1.00	0.85	0.90	0.94	0.77	1.00

^a^ Correlations around transcription start site are located in upper diagonal, while the correlations around transcription end sites are in the lower diagonal (see also Fig 2). Samples from Schmitz et al. [19] and Becker et al. [35] trans-generational studies.

^b^ The density of DMPs based on the absolute difference of methylation levels (which is equal to *TV*) estimated based on Fisher exact test.

A DMP detected by a particular divergence measure in Eq 29 (30) indicates that a statistically significant amount of energy was dissipated to produce it. But the amount of energy dissipated is relative to each tissue or individual. In addition, since the action of thermal fluctuations through the ontogenetic development of cells is not the same for every cell, DMPs may differ between lineages of identical genetic background. These biophysical aspects of the methylation process are addressed by the non-linear estimation of Eq 29 (30) for each individual, while these aspects are ignored by the application of statistical tests analogous or equivalent to Fisher’s exact test.

#### Differentially informative methylated regions (DIMRs)

Currently there are various methods used to define differentially methylated regions (DMRs) [34]. These approaches can be distinguished mainly into two classes: 1) those that rely on an algorithm for clustering genomic regions rich in DMPs, and 2) those that function on predefined genomic intervals [34]. The statistical mechanical model presented here can be applied to any of these variants. A sample hybrid method is given in the next section, where an approach for clustering of single cytosines is performed and followed by the estimation of the statistical mechanical CDF for the cluster of same size.

Now, let *π* be a subset of genomic regions of the same size. Then, for each element from *π*, an information-theoretic divergence *D*_*R*_ (Eq 28, e.g., Hellinger divergence *H*_*R*_) can be calculated and the cumulative distribution function estimated according to Eq 29. Then the definition of DMP given above is easily extended to define a differentially methylated region (DMR).

Let $P\left(E_{R}^{D}\leq E_{R}^{D_{0}}\right)$ be the probability that energy $E_{R}^{D}$, dissipated to create an observed divergence *D*_0_ between the methylation levels from two different samples at a given genomic region *R*, is lesser than or equal to the amount of energy $E_{R}^{D_{0}}$. Then, a single genomic region *R* represents a DMR at a level of significance *α* if, and only if, the probability $P\left(E_{R}^{D}>E_{R}^{D_{0}}\right)=1-P\left(E_{R}^{D}\leq E_{R}^{D_{0}}\right)$ to observe a methylation change with energy dissipation higher than $E_{R}^{D_{0}}$ is lesser than *α*.

As in the case of the definition of DMPs, probabilities $P\left(E_{R}^{D}\leq E_{R}^{D_{0}}\right)$ can be approached by giving specific values to the divergence *D*_*R*_ in Eq 28. For example, we can set $D_{R}={\hat{H}}_{R}^{D_{0}}$, where ${\hat{H}}_{R}^{D_{0}}$ is the Hellinger divergence estimated according to Eq 28 by making $D_{k}={\hat{H}}_{k}^{D_{0}}$, and ${\hat{H}}_{k}^{D_{0}}$ is estimated from the experimental data by Eq 27. Next, $P\left(E_{R}^{D}\leq E_{R}^{D_{0}}\right)≅P\left(H_{R}^{D}\leq {\hat{H}}_{R}^{D_{0}}\right)$ provided that $H_{R}^{D}$ is proportional to $E_{R}^{D}$, and probabilities $P\left(H_{R}^{D}\leq {\hat{H}}_{R}^{D_{0}}\right)$ can be estimated by means of Eqs 29 and (30), substituting *D*_*R*_ by ${\hat{H}}_{R}^{D_{0}}$ ($D_{R}={\hat{H}}_{R}^{D_{0}}$). A DMR detected based on an information divergence measure shall be called differentially informative methylated region (DIMR). In addition, it should be noticed that since the given definitions of DMPs and DMRs are based on the statistical mechanical CDF followed by the methylation background noise, there is an open door for the application of SDT and Bayesian SDT to bypass the limitations of current decision-making based on controversial *p*-values [29,36–40].

### The binary language of cytosine DNA methylation

**Fig 3:**
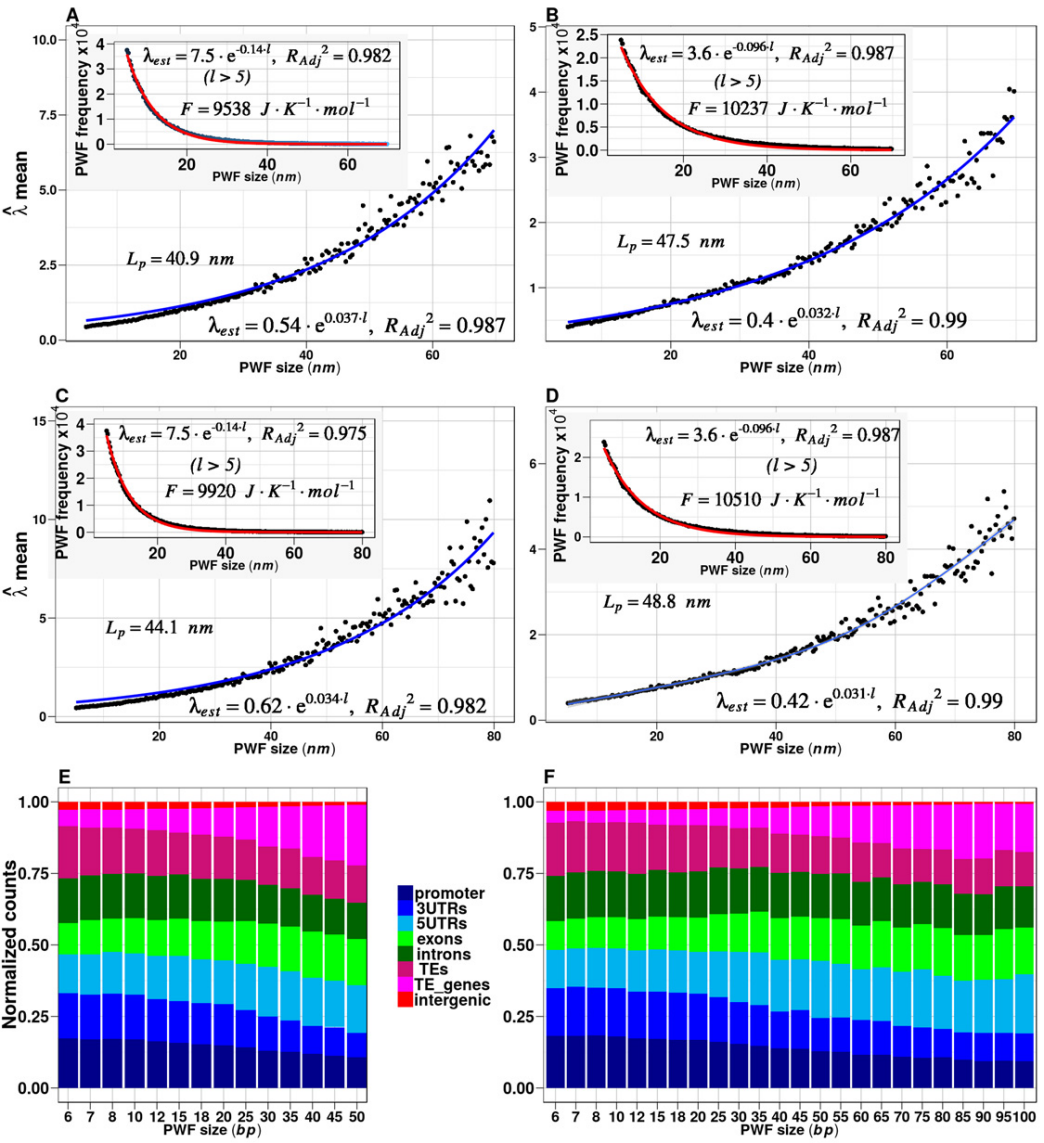
Statistical trends of $\hat{\lambda }\left(l\right)$ mean and *PWF* frequencies from partitions *S*_6_ and *S*_7_. Statistical trends were estimated in forty transgenerational methylome variants from *Arabidopsis thaliana* [19,35] considering all CDM contexts. (A) and (B), exponential region of $\bar{\hat{\lambda }}\left(l\right)$ *vs l* in the range of *PWF* from 5 to 70 nm for the partitions *S*_6_ and *S*_7_, respectively. (C) and (D), exponential regions of $\bar{\hat{\lambda }}\left(l\right)$ *vs l* in the range of *PWF* from 5 to 80 nm for the partitions *S*_6_ and *S*_7_, respectively. The exponential behavior is consistent with Eqs 22 and (23), which permits the estimation of the DNA persistence length *L*_*p*_ by means of Eq 24. (E) and (F) correspond to barplots for the annotated *PWFs* from linear regions presented in the panels A and B, respectively (4 to 100 bp ~ 34 nm). The relative frequencies of *PWFs* were normalized taking into account the overall length of the genomic region occupied by each genomic feature (promoter, exons, transposable elements (TEs), intergenic, etc) in the *Arabidopsis thaliana* genome. The exponential decay law predicted by Eq 33 was verified (subplots mean of *PWF*-*frequency* (*f*) *vs l* in panels A to D). The estimated value of the Helmholtz free energy Δ*F* = *RT lnZ*(*γ*) (Eq 34) at 298.15 K of temperature is indicated.

Results obtained may reflect the existence of a methylation language, with ‘words’ depicted in the binary alphabet of methylated (1) and non-methylated (0) bases. Postulating that the beginning and the end of a methylation word must be 1, genome-wide screening can be performed where two consecutive cytosine positions of value 1 are separated by less than a given threshold *d* of 0s. For a large enough methylome dataset, detection of the potential framework of letter variations is possible (see Materials and Methods). We have designated these variations as *Potential Word Frameworks* (*PWFs*). The results for a genome-wide screening in Arabidopsis that considers all cytosine methylation contexts are summarized in Fig 3. The analysis was limited to the case of the Weibull distribution given by Eq 14. GG distribution was not analyzed due to the high computational cost that conveys its nonlinear fit when the number of genomic regions goes over 50,000.

The exponential increment of the mean $\bar{\hat{\lambda }}\left(l\right)$ of $\hat{\lambda }\left(l\right)$ with the *PWF* length *l* is predicted by Eqs 22 and (23), which was derived after considering the mechanical behavior of small DNA fragments (with sizes *l* ≤ *L*_*p*_ or ∼*L*_*p*_) as a linear entropic spring that obeys Hooke’s law [25]. The nonlinear regression fit $\bar{\hat{\lambda }}\left(l\right)$ *vs l* permitted an alternative way to estimate the DNA persistence length ${\hat{L}}_{p}$ through Eq 24. The results are consistent with those reported in the literature (Fig 3 and S2 Fig for Arabidopsis ecotypes) [24–27]. Since ${\hat{L}}_{p}$ is a basic mechanical property of the DNA molecule that can be altered by CDM [10], this result and previous estimations for large genomic regions support the statistical mechanical basis of the Weibull distribution given by Eq 14. Consequently, Eq 14 can be properly used to determine whether or not a *PWF* is a DIMR. That is, Eq 14 can be used to discriminate methylation changes observed on a *PWF* that were created by the methylation regulatory machinery. This analysis provides a robust way for the *in silico* prediction of methylome “words”.

The exponential increment of the mean $\bar{\hat{\lambda }}\left(l\right)$ of $\hat{\lambda }\left(l\right)$ with the *PWF* length *l* (Fig 3) indicates the existence of an energetic limit for *PWF* size. According to Eqs 4 and 14, this observation implies a rapid decline in the probability of a methylation change with energy dissipation $E_{PWF_{l}^{d}}$ in a *PWF*_*l*_^*d*^ of length *l* derived from a methylome partition *S*_*d*_ into *PWFs* with threshold *d*. Moreover, a structured limit for *PWF* length seems to be encoded in the DNA sequence itself. The subplots in Fig 3 show an exponential decay in the frequency of *PWFs* with increasing length, so that the frequency of large *PWFs* is low. This exponential decay law (Eq 33) is consistent with the principles of maximum entropy and least effort. Probability distributions that minimize the costs per average bits of information contained in the *PWF*_*l*_*s* (Eq 32) are those with maximum Gibb-Shannon’s entropy (Boltzmann distributions). Thus, the enzymatic regulatory machinery, which “reads” the message carried by *PWF*_*l*_*s* and triggers the tissue response to environmental variation, receives, on average, the maximum amount of information at minimum cost. According to Eqs 14 and 33, *PWFs* cannot be arbitrarily large, and long *PWFs* represent “sentences” of shorter *PWFs* (see Material and Methods). Similar results for Arabidopsis ecotypes [41] are shown in S2 Fig.

The exponential decay in genomic frequency of *PWFs* with increasing size (Eq 33) establishes a thermodynamic restriction for binary methylation language. The average estimation of the Helmholtz free energy Δ*F* = *RT ln*(*N*_0_/*ϕ*) (Eq 35) could be a suitable indicator of the difference in methylation languages from different species. In particular, the estimations of Δ*F* for Arabidopsis samples from the trans-generational studies [19,35] and from ecotypes [41] (subplots in Fig 3 and S2 Fig, respectively) indicate that the Arabidopsis methylation language is stable at different environmental conditions. Indeed, the difference between corresponding estimations of Δ*F* reflects the expected natural variation within the limits of experimental and numerical error. If the Arabidopsis methylation language is consistent with thermodynamic theory used to derive Δ*F* = *RT lnZ*(*γ*) (Eq 34), then we must expect that, in a closed system at volume and temperature constants, Δ*F* is constant. In the current case, we are dealing with an open system and natural variation such as mutation can exist [41], implying variation of the system volume. Hence, in the Arabidopsis methylome dataset we must expect small natural variations of Δ*F*. This result points to a robust structure of the methylation language in Arabidopsis.

Within the current dataset, about 75% of *PWFs* comprise methylation signals concentrated in gene regions (Fig 3E and 3F). This finding in Arabidopsis is striking. It is believed that the three methylation contexts of CG, CHG and CHH may have distinct biological roles in Arabidopsis [1]. The primary genomic sites for differential methylation of contexts CHG and CHH are not gene regions, but more often transposable element and repetitive sequences. Although the algorithm for detection of *PWFs* does not make distinction between particular methylation contexts, the annotation of *PWFs* within gene regions indicates that the contribution derives largely from CG context. Learning the degree of specificity will require further refinement of *PWFs* and more detailed experimental confirmation. For example, a particular $PWF_{11}^{6}$ with eleven digits from a methylome partition *S*_6_ into *PWFs* with threshold *d* = 6 could be 11010000011 (subject to a given data set), while a particular realization of this *PWF* could be 11110000011. Such results would suggest the existence of an epigenomic code, or a set of methylation rules that determine whether or not a binary stretch of methylation marks is a meaningful signal for recognition by the molecular machines that trigger tissue response.

## Conclusions

Results to date encompass the classical methylome analysis based on DMP detection, and suggest that CDM underlies an epigenomic communication system in living tissues, shown here in *Arabidopsis thaliana* and human samples. These observations present an approach for epigenomic studies within a framework of communication systems. The information thermodynamic modeling proposed here unveils links between genome-wide methylation analysis, molecular thermodynamics and information theory. The application of an information thermodynamics approach permits not only the discrimination of biological signal from methylation background noise, but also the application of Bayesian SDT [29,36]. That is, application of Bayesian SDT together with the information thermodynamics process provides the formulae for robust detection of epigenetic biomarkers [29,38,39].

We describe here an open problem to be confronted by the application of coding theory, a means of estimating the code-words that would maximize error control in an epigenomic communication system (see [42] for a brief overview). Digital signal processing (DSP) provides the tools to analyze genome-wide regulatory features of such an epigenomic signal [43]. Potential applications of coding theory, DSP and SDT in a multifaceted, reiterative process should ultimately lead to successful deciphering of the epigenomic code. Knowledge of such a methylation code would create new opportunities with important biomedical and agricultural implications.

## Material and Methods

### Information processed by the methylation machinery in a genomic region

The addition or removal of a methyl group to a cytosine C5 residue within a DNA molecule can be verified by DNA bisulfite conversion methodology coupled with next-generation sequencing approaches (Bis-seq), allowing determination of the methylation status of nearly every cytosine in a genome. Methylation status of particular cytosine sites is then expressed in terms of methylation level *p*_*i*_ = #*C*_*i*_/(#*C*_*i*_ +#*nonC*_*i*_), where #*C*_*i*_ and #*nonC*_*i*_ represent the numbers of methylated and non-methylated read counts observed at the genomic coordinate *i*, respectively. At a tissue level, methylation status (methylated or non-methylated) of cytosine *C*_*i*_ at the genomic coordinate *i* can be analyzed as a random variable that takes value “methylated” with probability *p*_*i*_ and “non-methylated” with probability 1 − *p*_*i*_.

Shannon’s entropy *H*(*p*(*x*_*i*_)) = −∑_*i*_ *p*(*x*_*i*_)*log*_2_*p*(*x*_*i*_) (1) of a random event with probability distribution *p*(*x*_*i*_) has been widely accepted as a measure of the uncertainty associated with random events [44]. In particular, an expression similar to Eq 1 was used in an experimental demonstration of information-to-energy conversion [45]. A modified expression of Eq 1 has been applied to quantitatively assess the variation in DNA methylation patterns [46]. The inherent uncertainty of the methylation status at each cytosine site leads to the direct application of Eq 1 to experimental data obtained from plant and animal tissues:

$$H\left(C_{i}\right)=-p\left(C_{i}\right)log_{2}p\left(C_{i}\right)-\left(1-p\left(C_{i}\right)\right)log_{2}\;\left(1-p\left(C_{i}\right)\right)$$ (2)

The entropy defined by Eq 2 is therefore the expected value of the logarithm base 2 of the methylation level [47].

Assuming that, as a result of variations in environmental conditions, a change of methylation status in genomic region *R* takes place, the absolute amount of information processed by the methylation machinery in the genomic region *R* is given by:

$$I_{R}=\left|\;\sum_{i\in R}H\left(C_{i}^{after}\right)-\sum_{i\in R}H\left(C_{i}^{before}\right)\right|$$ (3)

Where $C_{i}^{before}$ and $C_{i}^{after}$ stand for the methylation status before and after the variations of environmental conditions, respectively. That is, the absolute amount of information *I*_*R*_ is defined as the absolute difference between two entropies (the uncertainty change) associated with the knowledge about two states (before and after) of a given system [22,48,49]. At tissue or organ levels, Eq 3 gives the uncertainty variation of the methylation status originated by the methylation changes at a given genomic region *R* or a single cytosine site.

### Derivation of probability density functions (PDF) and cumulative distribution functions (CDF) for energies *E*_*R*_ and information *I*_*R*_

A methylation change at a genomic region *R* has an associated amount of information *I*_*R*_ processed by the activity of methyltransferases and demethylases. To estimate the amount of information associated with methylation changes, a methylome is split into *N* genomic regions of length *l*, and information *I*_*R*_ is computed according to Eq 3 in each region *R*. Under Landauer’s principle, the minimum energy dissipated to process the information *I*_*R*_ can be approached by equation: *E*_*R*_ = *I*_*R*_*k*_*B*_ *T ln*2 (4).

For a fixed length of genomic region *R*, the range of possible values for energy dissipation *E*_*R*_ along a methylome is large, but with a finite range of possible values. We assume that methyltransferase/demethylase activities at different genomic regions are independent of one another, and that the methylation changes induced by the action of thermal fluctuation are independent as well. Kinetic parameters and mechanisms of enzymatic reaction catalyzed by methyltransferases are assumed to be consistent across different genomic regions.

Derivation of the generalized gamma (GG) distribution follows the derivation given by Lienhard and Meyer [50], with the assumptions rewritten for the context of cytosine DNA methylation (CDM). Let *N*_*i*_ be the number of time that an amount of energy in the interval $\left[E_{R}^{i-1},E_{R}^{i}\right)$ is dissipated in *N* genomic regions (GRs). The following requirements are imposed upon *N*_*i*_:

1. The total number of occurrence of the event is fixed: ∑_*i*_*N*_*i*_ = *N*; *N*_*i*_ ’s and *N* are assumed large numbers.
2. For each choice of *δ* the following sum is a positive constant: $\sum_{i}\frac{N_{i}}{N}{\left(E_{R}^{i}\right)}^{\delta }=K$
3. The number of distinguishable ways, *n*_*i*_, in which the event can occur with values in the interval $\left[E_{R}^{i-1},E_{R}^{i}\right)$ is proportional to a specific power of $E_{R}^{i}$. That is, $n_{i}=A{\left(E_{R}^{i}\right)}^{\nu -1}$.

In addition, *δ*, *ν*, and *K* > 0. Assumption 3 can be derived from physical constraints (S1 Appendix, Eqs 2 and 3), but here we are following Lienhard and Meyer [50] derivation. Under these assumptions, the reasoning indicated by Lienhard and Meyer [50], leads to the GG distribution with parametrization given by Stacy [51]:

$$f\left(E_{R}|a,\delta ,\nu \right)=\frac{\delta }{a^{\nu }\Gamma \left(\nu /\delta \right)}E_{R}^{\nu -1}\;e^{-{\left(\frac{E_{R}}{a}\right)}^{\delta }}$$ (5)

The form commonly used in practice is obtained by the parametrization: *ψ* = *ν*/*δ*, *β* = *a*, and *α* = *δ*:

$$f\left(E_{R}|\alpha ,\beta ,\psi \right)=\frac{\alpha }{\beta \;\Gamma \left(\psi \right)}{\left(\frac{E_{R}}{\beta }\right)}^{\alpha \psi -1}\;e^{-{\left(\frac{E_{R}}{\beta }\right)}^{\alpha }}$$ (6)

With a scale parameter *β*, and two shape parameters, *α* and *ψ*. After splitting a methylome into relatively large genomic regions, it is possible that every region contains at least one or more methylation changes in such a way that *E*_*R*_ > *η* > 0 for all regions *R*. From a statistical point of view, *η* is a location parameter and, in this case, the last equation adopts the form:

$$f\left(E_{R}|\alpha ,\beta ,\eta ,\psi \right)=\frac{\alpha }{\beta \;\Gamma \left(\psi \right)}{\left(\frac{E_{R}-\eta }{\beta }\right)}^{\alpha \psi -1}e^{-{\left(\frac{E_{R}-\eta }{\beta }\right)}^{\alpha }}$$ (7)

Since methylation changes can take place with random fluctuations in thermal noise, the scaling parameter *β*(*l*) can be set equal to the average energy per DNA molecule in thermal equilibrium. That is, *β*(*l*) = *φ*(*l*)*k*_*B*_*T* (8), where *φ*(*l*) expresses the contribution of all degrees of freedom to the average energy per molecule as a function of genomic region length *l*.

Under the Landauer principle, we can use Eq 4 to derive the probability density function of the information *I*_*R*_, which is also a GG distribution:

$$f\left(I_{R}|\alpha ,\lambda ,\mu ,\psi \right)=\frac{\alpha }{\lambda \left(l\right)\;\Gamma \left(\psi \right)}{\left(\frac{I_{R}-\mu }{\lambda \left(l\right)}\right)}^{\psi \alpha -1}e^{-{\left(\frac{I_{R}-\mu }{\lambda \left(l\right)}\right)}^{\alpha }},I_{R}>\mu >0$$ (9)

Where *λ*(*l*) = *φ*(*l*)/*ln* 2 (10) and *μ* = *η*/(*k*_*B*_ *T ln* 2) (11) is the location parameter of the GG distribution of *I*_*R*_.

The cumulative distribution function for Eq 9 is given by:

$$F\left(I_{R}|\alpha ,\mu ,\lambda \left(l\right),\psi \right)=\{\begin{array}{l} \;\frac{1}{\Gamma \left(\psi \right)}\gamma \left(\psi ,{\left(\frac{I_{R}-\mu }{\hat{\lambda }\left(l\right)}\right)}^{\alpha }\right)\;\alpha >0\;\text{and}\;I_{R}>\mu >0\\ 1-\frac{1}{\Gamma \left(\psi \right)}\gamma \left(\psi ,{\left(\frac{I_{R}-\mu }{\lambda \left(l\right)}\right)}^{\alpha }\right)\;\alpha <0\;\text{and}\;I_{R}>\mu >0 \end{array}$$ (12)

Where *γ*(⋅) denotes the lower incomplete gamma function. GG distribution gives rise to a family of distributions, which encompasses Weibull, gamma, Rayleigh, exponential, and Maxwell velocity distributions and lognormal as a limiting distribution [50]. An extended list of members of this family of distributions can be found in Crooks [52]. In particular, Weibull distributions with PDF: $f\left(I_{R}|\alpha ,\lambda ,\mu \right)=\frac{\alpha }{\lambda \left(l\right)}{\left(\frac{I_{R}-\mu }{\lambda \left(l\right)}\right)}^{\alpha -1}\;e^{-{\left(\frac{I_{R}-\mu }{\lambda \left(l\right)}\right)}^{\alpha }},I_{R}>\mu >0$ (13) derives from Eq 9 when *ψ* = 1. Weibull CDF is given by $F\left(I_{R}|\alpha ,\lambda ,\mu \right)=1-e^{-{\left(\frac{I_{R}-\mu }{\lambda \left(l\right)}\right)}^{\alpha }}$ (14). The parameters from Eqs 12 and 14 can be estimated from the nonlinear fit of *I*_*R*_ values computed from the experimental methylome data at the different fixed windows of length *l* used to split the genome into non-overlapping genomic regions.

Under Landauer’s principle, Eqs 9–14 must hold; likewise for any member of a GG distribution family derived as a particular case of Eq 9.

#### Physics of the scaling parameter

The scaling parameters *β*(*l*) and *λ*(*l*) are expressed in terms of *φ*(*l*), which is a function of the contribution of all degrees of freedom to the average energy per molecule *E*(*l*) and, ultimately, a function of the counter length *l* for the DNA regions under consideration. For a large genomic region, the complexity of motion complicates a full theoretical derivation of an expression for *φ*(*l*).

A double-stranded DNA (dsDNA) molecule in solution bends and curves locally as a result of thermal fluctuations [25]. In the inextensible worm-like model, the molecule is treated as a flexible rod of length *l* that curves smoothly. One assumes a general exponential relationship *λ*(*l*) *vs E*(*l*): $\lambda \left(l\right)=a\;e^{\frac{E\left(l\right)}{\frac{1}{2}k_{B}T}}$ (15), where *a* is a proportionality constant. The simplest approach assumes *E*(*l*) proportional to $\frac{l}{L_{p}}$ (for *l* >> *L*_*p*_), i.e., $E\left(l\right)=c\frac{l}{L_{p}}$ where *L*_*p*_ is the persistence length of the DNA molecule (~ 50 nm, [24,53]) and *c* is a proportionality constant. Next, we can set $\lambda \left(l\right)=a\;e^{\frac{b\;l}{L_{p}}}$ (16), where $b=\frac{2c}{k_{B}T}$. Taking the first two terms of the Taylor expansion of Eq 16 around *L*_*p*_ gives: $\lambda \left(l\right)≅\left(1-b\right)a\;e^{b}+\frac{a\;e^{b}b}{L_{p}}l$ (17). Taking into account Eq 10, we can set *a e*^*b*^ = (*ln* 2)^−1^ and rewrite Eq 16 as $\lambda \left(l\right)=\frac{\left(1-b\right)}{ln\;2}+\frac{b}{L_{p}ln\;2}l$ (18). Under Landauer’s principle, the empirical averages $\bar{\hat{\lambda }}\left(l\right)$ of $\hat{\lambda }\left(l\right)$ estimations obtained for each set of methylome data (e.g., Arabidopsis ecotypes and human cell tissues) must not be statistically different from the estimations $\hat{\lambda }{\left(l\right)}_{est}$ obtained from linear regression analyses $\bar{\hat{\lambda }}\left(l\right)$ versus *l*. This regression analysis yields the equation of a straight line λ^est(l)=a^'+b^'l (19), where a^'=(1−b^)ln2 and b^'=b^L^pln2. An estimation of the DNA persistence length ${\hat{L}}_{p}$ from the experimental data can be obtained combining the last two equations L^p=1−a^'ln2b^'ln2 (20).

For short genomic regions, with sizes on the order of *L*_*p*_, a DNA fragment is bent only by a small amount, while for *l* << *L*_*p*_ it is essentially straight. However, dsDNA behaves as a linear entropic spring with a Hooke’s constant $k_{DNA}=\frac{3k_{B}T}{2L_{p}l}$ [25]. Once compressed by thermal forces, the DNA can be stretched to a distance close to *l*. The spring energy that equates to this thermal energy is given by $E\left(l\right)=\frac{1}{2}k_{DNA}\;l^{2}$, or more precisely, $E\left(l\right)=\frac{3k_{B}T}{4L_{p}}\;l$ (21). The substitution of the last equation into Eq 15 yields: $\lambda \left(l\right)=a\;e^{\frac{3}{2}\frac{l}{L_{p}}}$ (22). Thus, under Landauer’s principle, for short DNA fragments, the nonlinear regression analysis $\bar{\hat{\lambda }}\left(l\right)$ versus *l* yields the exponential equation: ${\hat{\lambda }}_{est}\left(l\right)=\hat{a}\;e^{\hat{d}\;l}$ (23), where $\hat{d}=\frac{3}{2{\hat{L}}_{p}}$, providing another means for experimental estimation of the DNA persistence length: ${\hat{L}}_{p}=\frac{3}{2\hat{d}}$ (24).

To estimate the parameters from experimental data in Arabidopsis ecotypes and human tissues, each methylome was split into genomic regions of fixed length *l*, from 2000 to 5000 bp (this can be done, for example, with the R function “*tileGenome*” from the R package “*GenomicRanges*”). In the case of Arabidopsis trans-generational samples, *PWF*s (short DNA regions, see below) were used as the genomic intervals.

### Divergence between the methylation levels

According to Eq 3, the uncertainty variation at a single cytosine position is zero when methylation levels go from 0 to 1 (or vice versa). At a tissue level, there is no gain or loss of information, and all cells in the tissue are synchronized for epigenetic response. However, we seek to discriminate between these methylation levels and to evaluate whether or not a cytosine position would be linked to a specific epigenetic response.

Alternative information-theoretical measures can be applied to estimate *divergence* between methylation levels from two samples. Three additional information-theoretic measures were considered: Total-variation (*TV*), Kullback–Leibler (*KL*) and Hellinger (*H*^*D*^) divergences. *TV* is the absolute value of the difference of methylation levels. *KL* gives the maximum information one might gain by observing a system [22], although the extreme methylation change from 0 to 1 (and vice versa) has zero gain or loss of information. *H*^*D*^ is able to discriminate between all methylation levels. At a single cytosine position, *TV*, *KL* and *H*^*D*^ are computed by the expressions *TV*(*p*, *q*) = |*p* − *q*| (25), $KL\left(p,q\right)=p\;log\frac{p}{q}+\left(1-p\right)\;log\frac{1-p}{1-q}$ (26) and $H^{D}\left(p,q\right)={\left(\sqrt{p}-\sqrt{q}\right)}^{2}+{\left(\sqrt{1-p}-\sqrt{1-q}\right)}^{2}$ (27), where *p* and *q* are the methylation levels of the samples under comparison. It is known that *TV* ≤ *H*^*D*^ ≤ *KL* ≤ *χ*^2^, where *χ*^2^ is the chi-squared divergence, also known as Pearson's chi-squared statistic. That is, *H*^*D*^ provides a conservative criterion for the divergence between the methylation levels.

A definition of *D*_*R*_ can be given as in the case of *I*_*R*_ in Eq 3: *D*_*R*_ = ∑_*k*∈*R*_ *D*_*k*_ (28).

Next, let $E_{R}^{D}$ be the energy dissipated to create the observed divergence *D*_*R*_ between methylation levels at the genomic region *R*. All the assumptions and constraints applied to deduce the PDF of *E*_*R*_ given by Eq 10 hold for $E_{R}^{D}$. Thus, $E_{R}^{D}$ must follow a GG distribution or a Weibull distribution or some distribution from the GG distribution family. In consequence, an information-theoretical measure *D*_*R*_ applied to express divergence between the methylation levels will follow Weibull distributions, provided that *D*_*R*_ is proportional to $E_{R}^{D}$. Therefore,

$$F\left(D_{R}|\;\alpha ,\mu ,\lambda \left(l\right),\psi \right)=\{\begin{array}{l} \;\frac{1}{\Gamma \left(\psi \right)}\gamma \left(\psi ,{\left(\frac{D_{R}-\mu }{\hat{\lambda }\left(l\right)}\right)}^{\alpha }\right)\;\alpha >0\;\text{and}\;D_{R}>\mu >0\\ 1-\frac{1}{\Gamma \left(\psi \right)}\gamma \left(\psi ,{\left(\frac{D_{R}-\mu }{\lambda \left(l\right)}\right)}^{\alpha }\right)\;\alpha <0\;\text{and}\;D_{R}>\mu >0 \end{array}$$ (29)

From where we could derive the CDF of any member of the GG distribution family, i.e., for the Weibull distribution $F\left(D_{R}|\;\alpha ,\lambda ,\mu \right)=1-e^{-{\left(\frac{D_{R}-\mu }{\lambda \left(l\right)}\right)}^{\alpha }}$ (30), where the parameters *λ*, *α* and *μ* play analogous role to those found in Eq 14.

### Detection of Potential Word Frameworks (PWFs)

To detect *PWFs*, a long string of zeros and ones was derived from the *GRanges* object in R [54] containing the methylome samples. This string was built according to the following criterion: if the methylation level for a given cytosine position was greater than zero in at least one methylome, then a numerical value of 1 was assigned to that position, otherwise, the value 0 was assigned. The string was divided into clusters taking into account that the value 1 must be found at the beginning and end of a *PWF*, and *S*_*d*_ denotes the partition of the methylome into clusters derived after fixing threshold *d* with a particular value.

Any cluster from *S*_*d*_ will be a *PWF* or a string integrated only by zeros. We use the symbols $s_{l}^{0}$ and $PWF_{l}^{d}$ to denote a string with *l* zeros and a *PWF* of length *l* from partition *S*_*d*_, respectively. As a result, for any partition *S*_*d*_ with *d* ≥ 2, a $PWF_{l}^{d}$ is the union of $PWF_{i}^{k}$ from partitions *S*_*k*_ and strings $s_{l}^{0}$ for which *k* < *d* − 1 and *l* = *d* − 1. That is, a $PWF_{l}^{d}$ from partition *S*_*d*_ can be considered as a sentence formed by words from partitions *S*_*k*<*d*−1_ and strings $s_{l=d-1}^{0}$. For example, $PWF_{28}^{7}=\underset{PWF_{5}^{3}}{\underset{︸}{10011}}\underset{s_{6}^{0}}{\underset{︸}{000000}}\underset{PWF_{9}^{6}}{\underset{︸}{100000111}}\underset{s_{6}^{0}}{\underset{︸}{000000}}\underset{PWF_{2}^{1}}{\underset{︸}{11}}$ denotes a sentence integrated by *PWFs* from *S*_3_, *S*_6_, and *S*_1_ and two strings of zeros $s_{6}^{0}$. A value zero at a given cytosine position does not mean that the cytosine cannot be methylated, but that the frequency is very low. The bias originating from cytosine sites with low methylation frequencies is adjusted by increasing the number of methylomes included in the analysis. Our present analyses were limited to two independent datasets: 40 trans-generational methylome variations of Arabidopsis and 152 methylomes from Arabidopsis ecotypes.

The amount of information *I*_*R*_ was estimated for each $PWF_{l}^{d}$ of length *l* from partition *S*_*d*_. The corresponding $PWF_{l}^{d}$ from different methylomes were pooled to one set. The algorithmic approach used here is simple, but does not preclude alternative approaches. Next, a non-linear fit of Eq 14 was performed with the set of $PWF_{l}^{d}$ from each methylome and the estimations of $\hat{\lambda }\left(l\right)$ were used to compute ${\hat{L}}_{p}$ according to Eqs 23 and 24, as described above.

### Relationship between the genomic frequency and the length of PWFs

We denote by *C*_*l*_ the energetic cost of establishing a *PWF*_*l*_ of length *l*, and let *C* = ∑_*l*_ *p*_*l*_ *C*_*l*_ (31) represent the average energetic cost per *PWF*, where *P* = {*p*_*l*_} is the probability distribution of *C*_*l*_. The distribution *P* = {*p*_*l*_} can be determined that satisfies the constraint given by Eq 31 and has the highest Gibb-Shannon’s entropy. This solution minimizes the average cost per average bit of information contained in the *PWF*_*l*_*s* detected by the regulatory methylation machinery: *γ* = *C*/*H*, where *H* = *H*(*PWF*_*l*_) is given by Eq 1. Under these assumptions, the only solutions are the Boltzmann distributions $p{\;}_{l}^{*}\left(\beta_{0}\right)=e^{-\beta_{0}\;C_{l}}/Z\left(\beta_{0}\right)$ (32) with parameter *β*_0_ and *canonical partition function* of the system $Z\left(\beta_{0}\right)=\sum_{l}e^{-\beta_{0}\;C_{l}}$ [44]. The frequencies *f*_*l*_ of *PWF*_*l*_ follow an exponential decay law with the increment of length *l*, provided that *C*_*l*_ = *cl*, where *c* is a constant of proportionality, i.e., *f*_*l*_(*l*/*γ*, *N*_0_) = *N*_0_*e*^−*γl*^/*Z*(*γ*) or *f*_*l*_(*l*/*γ*, *ϕ*) = *ϕe*^−*γl*^ (33), where *ϕ* = *N*_0_/*Z*(*γ*), *γ* = *β*_0_*c*, and *N*_0_ is the total number of *PWFs* in a given partition *S*_*d*_ of the methylome. Since the value of *N*_0_ can be estimated from the experimental data, we can estimate the value of *lnZ*(*γ*), where *β*_0_ = (*k*_*B*_*T*)^−1^ (or *β*_0_ = (*RT*)^−1^ and *R* is the gas constant) leads us to an estimation of the Helmholtz free energy Δ*F* = *k*_*B*_*T ln Z*(*γ*) or Δ*F* = *RT ln Z*(*γ*) (34) that measures maximum “useful” work obtainable from the closed thermodynamic system at a constant volume and temperature. In the present case, Δ*F* = *RT ln*(*N*_0_/*ϕ*) (35).

### Arabidopsis methylation data

According to Eq 3, *I*_*R*_ is computed for a subject sample with respect to a given reference sample. The *I*_*R*_ values were computed for 150 Arabidopsis ecotypes [41]. The TSV files taken from NCBI GEO under accession GSE43857 [41] were read and transferred to R software version 3.2.1 [54] by using the Bioconductor (version 2.14) R-package *GenomicFeatures* [55]. Ecotype Col-0 was used as reference (151 ecotypes including Col-0). In addition, forty BS-seq samples from Schmitz et al. [19] and Becker et al. [35] trans-generational studies were analyzed. For these samples, the BS-seq reads from Fastq files were aligned to the TAIR10 genome with BSMAP allowing two mismatches. Methylation ratios were determined using a Python script (methratio.py) distributed together with the BSMAP software [56]. In the last case, a sample from the third generation from each study was taken as reference to compute *I*_*R*_.

### Human cell tissue methylation data

The *I*_*R*_ values were computed for 94 methylomes of human cell tissues taken from the NCBI GEO database. Data samples in “wig” format were read and processed by R software version 3.1.1 [54] with the Bioconductor R-packages *rtracklayer* and *GenomicFeatures* [55,57]. GEO accession numbers are given in S2 Table. The methylome of the undifferentiated embryonic stem cell line UCSF-4 (A21771-1, GSM1127122) was used as reference.

When multiple methylome data are analyzed simultaneously, coverage (#*C*_*i*_ +#*nonC*_*i*_) for the same cytosine site across the samples is not always available. Normally, missing data arise during the experimental workflow. Some samples were missing data at a given position while remaining samples preserved the information. We did not consider it advisable to remove these sites from our analysis. Since, by definition the entropy of zero is considered zero, replacement of the missing data by zero does not affect the calculus performed using Eq 3. So, for each set of methylomes (*Arabidopsis* and humans) samples were arranged into a unique *GRanges* object (R-package *GenomicFeatures*) used as the starting dataset of our computations.

### Statistical analyses

Statistical analyses were performed with R [54]. For each methylome, the parameters of Eq 5 were estimated by applying the Levenberg-Marquardt nonlinear least-squares algorithm available in R-package *minpack*.*lm*. Cross-validations for the nonlinear regressions were performed in each methylome as previously described [58]. In addition, Stein’s formula for adjusted R squared ($R_{Adj}^{2}$) was used as an estimator of the average cross-validation predictive power [58]. The main results from the statistical analyses are available as Supporting Information (S1–S6 Datasets).

## Supporting Information

S1 Appendix Alternative derivations of Weibull PDF and CDF for energies *E*_*R*_ and information *I*_*R*_. (PDF)

S1 Dataset Coordinates of the *PWFs* from partitions *S*_6_. (ZIP)

S2 Dataset Coordinates of the *PWFs* from partitions *S*_7_. (ZIP)

S3 Dataset Annotation of the *PWFs* from partitions *S*_6_. (ZIP)

S4 Dataset Annotation of the *PWFs* from partitions *S*_7_. (ZIP)

S5 Dataset Non-linear regression results for *PWFs*. (ZIP)

S6 Dataset R-scripts used in S1 Appendix. (ZIP)

S1 Fig Histograms, density plots and PP-plots for the Arabidopsis ecotypes Seattle-0 and Fr-2 at two different genomic region sizes. The empirical CDF of *I*_*R*_ departs from the theoretical CDF given in Eq 14 for genomic region sizes *l* below 2 Kb. The analysis of the PDFs and the CDFs reveals a significant increase in the frequency of genomic regions with very small information changes (*I*_*R*_ values close to zero) as the methylome is split into regions with sizes *l* < 2 Kb. PDF curves corresponding to the theoretical parameters estimated from Eq 14 (blue), and kernel density estimations (e.g.,“empirical” estimations that depend on the algorithm, kernel and bandwidth used) are also shown (black).
(TIF)

S2 FigStatistical trends of $\hat{\lambda }\left(l\right)$ mean and *PWF* frequencies from partitions *S*_6_ and *S*_7_.Statistical trends were estimated in 150 Arabidopsis ecotypes [41] considering all CDM contexts. (A) and (B), exponential region of the relationship $\bar{\hat{\lambda }}\left(l\right)$ *vs l* in the range of *PWF* from 5 to 70 nm for the partitions *S*_6_ and *S*_7_, respectively. (C) and (D), exponential regions of $\bar{\hat{\lambda }}\left(l\right)$ *vs l* in the range of *PWF* from 5 to 80 nm for the partitions *S*_6_ and *S*_7_, respectively. The exponential behavior is consistent with Eq 22 (23), which permits the estimation of the DNA persistence length *L*_*p*_ by means of Eq 24. The exponential decay law predicted by Eq 33 was verified (subplots mean of *PWF*-*frequency* (*f*) *vs l* in panels A to D). The estimated value of the Helmholtz free energy Δ*F* = *RT ln Z*(*γ*) (Eq 34) at 298.15 K of temperature is indicated.(TIF)

S1 Table Results of the non-linear fit of Eq 14 for 150 methylomes of *Arabidopsis* ecotypes. The non-linear fit was performed for the genomic regions from 2, 2.5, 3, 3.5, 4, 4.5, and 5 Kb (CG methylation context).
(XLSX)

S2 Table Results of the non-linear fit of Eq 14 for the sets of 80 human cell tissues. The non-linear fit was performed for genomic regions from 2, 2.5, 3, 3.5, 4, 4.5, and 5 Kb (CG methylation context).
(XLSX)
