# Supplementary material for: Information Thermodynamics of Cytosine DNA Methylation
Source: PLoS One. 2016 Mar 10;11(3):e0150427. doi: 10.1371/journal.pone.0150427 (PMC4786201; doi:10.1371/journal.pone.0150427)
Supplement: S1 Appendix — (PDF) [file pone.0150427.s001.pdf]

Appendix S1. Alternative derivations of Weibull PDF and CDF for energies E_R and data testing

General assumptions

A methylation change at a genomic region R has an associated amount of information I_R processed by the activity of methyltransferases and demethylases. To estimate the amount of information associated with methylation changes, a methylome is split to N genomic regions of length l, and information I_R is computed according to Eq. 3 in each region R.

The general assumptions for the model are:

1) Landauer's principle is assumed to hold. That is, under Landauer's principle, the minimum energy dissipated to process the information I_R can be approached by equation $E_R = I_R k_B T \, ln 2$ (Eq. 4, main text).
2) Methyltransferase/demethylase activities at different genomic regions are independent of one another. In addition, kinetic parameters and mechanism of enzymatic reaction catalyzed by methyltransferases are assumed to be consistent across different genomic regions.
3) Cytosine DNA methylation (CDM) changes induced by thermal fluctuations in non-overlapping genomic regions are independent for all the genomic regions.
4) There is a large, but finite, range of possible values of energy dissipation and any amount of energy $E_R \in \left[E_R^{i-1}, E_R^{i}\right)$ in a small interval of values $\left[E_R^{i-1}, E_R^{i}\right)$ is dissipated with constant probability $q << 1$.

Derivation assuming a Binomial process

We assume that the dissipation of each particular value of energy E_R follows a binomial process. In consequence, if the energy E_R associated to the CDM changes induced by thermal fluctuations is consistent with a binomial process (assumption 3 and 4), then we can distinguish these CDM changes from those originating by the regulatory methylation machinery (the biological signal), because it is well known that the latter are not independent for all genomic regions. Under this assumption, the probability that a particular value of energy E_R in the range $\left[E_R^{i-1}, E_R^{i}\right)$ would be dissipated at least once in N genomic regions is given by the binomial distribution $B(1, N, q) = N\,q\,(1-q)^{N-1}$.

Next, a natural statistical mechanical assumption considers the frequencies $f\left(E_R\right)$ proportional to the Boltzmann factor $e^{-\left(\frac{E_R}{\beta}\right)}$, i.e., $f\left(E_R\right) \propto e^{-\left(\frac{E_R}{\beta}\right)}$, where β is a scaling parameter. The Boltzmann factor, $e^{-\left(\frac{E_R}{\beta}\right)}$ reveals the relative probability of a particular arrangement (with a given energy). The experimental data confirm the last assumption (see below Fig.1 and 2). Hence, $f\left(E_R\right) = \frac{\alpha}{\beta} N q (1-q)^{N-1} e^{-\left(\frac{E_R}{\beta(l)}\right)}$ (1), where α is a proportionality constant. According to assumption 5, the approximation $(1-q)^{N-1} \cong e^{-Nq}$ can be used and we can rewrite Eq. 1 as: $f\left(E_R\right) = \frac{\alpha}{\beta} N q e^{-Nq} e^{-\left(\frac{E_R}{\beta(l)}\right)}$ (1), where Nq is the expected number of times that an amount of energy $E_R \in \left[E_R^{i-1}, E_R^{i}\right)$ can be dissipated in N genomic regions $\nu = Nq$ factor $e^{-\left(\frac{I_R}{\beta(l)}\right)}$. Under Landauer's principle $E_R = I_R k_B\, T\, ln\, 2$ and, thus, $f\left(E_R\right) \propto e^{-\left(\frac{E_R}{\beta(l)}\right)}$ must hold .

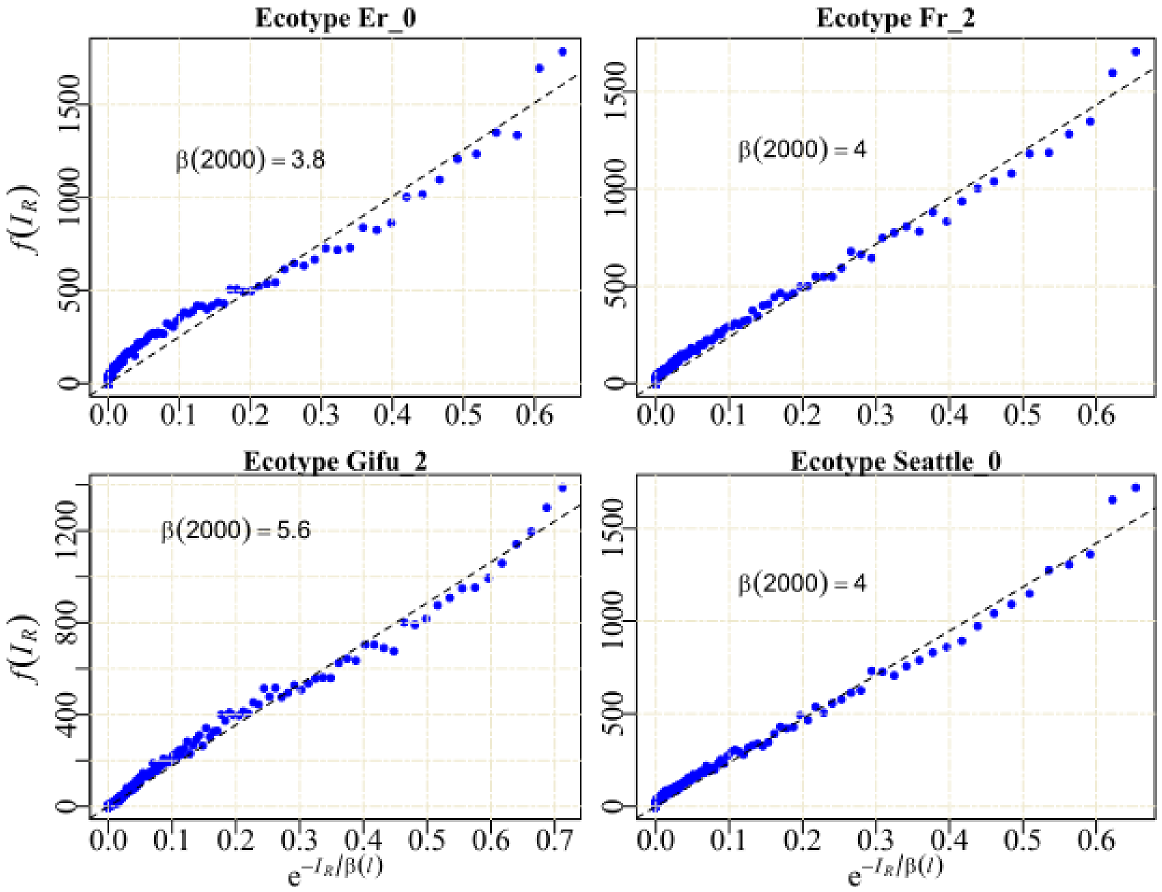

Figure 1. Relationship between $f\left(I_R\right)$ and Boltzmann factor $e^{-\left(\frac{I_R}{\beta(l)}\right)}$ for four Arabidopsis *thaliana* ecotypes with a methylome partition into non-overlapping windows of 2000 bp. Experimental data indicate that frequencies $f\left(I_R\right)$ are proportional to Boltzmann factor $e^{-\left(\frac{I_R}{\beta(l)}\right)}$. Under Landauer's principle $E_R = I_R k_B T\, ln 2$ and, thus, $f\left(E_R\right) \propto e^{-\left(\frac{E_R}{\beta(l)}\right)}$ must hold.

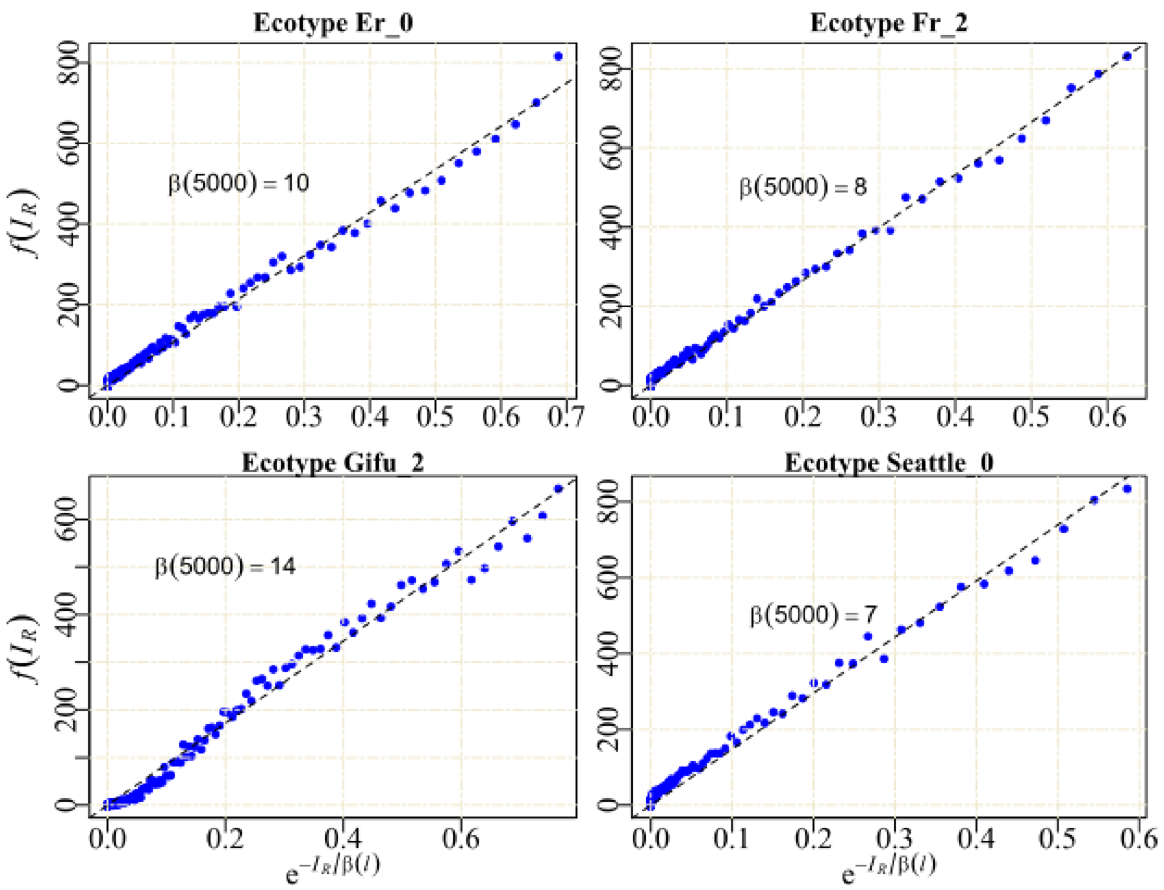

Figure 2. Relationship between $f(I_R)$ and Boltzmann factor $e^{-\left(\frac{I_R}{\beta(l)}\right)}$ for four Arabidopsis *thaliana* ecotypes with a methylome partition into non-overlapping windows of 5000 bp. Experimental data indicate that frequencies $f(I_R)$ are proportional to Boltzmann factor $e^{-\left(\frac{I_R}{\beta(l)}\right)}$. Under Landauer's principle $E_R = I_R k_B T \ln 2$. Thus, $f(E_R) \propto e^{-\left(\frac{E_R}{\beta(l)}\right)}$ must hold.

In nature, high-energy dissipation values E_R imply the processing of a considerable amount of information that, in the current case, conveys many methylation changes in the genomic region *R*. Massive methylation changes have been observed under extreme stress conditions or by mutation of a crucial gene for the methylation machinery. So, it is expected that, under normal conditions, high values of energy E_R are dissipated with low probability *q*. Then, q can be estimated subject to the constraint $\ln(q) = (\alpha - 1)\ln\left(\frac{E_R}{\beta(l)}\right) + c(l)$ ($E_R > 0$) (2), where $c(l)$ is a constant parameter that depends on the genomic region size l. Equation 2

leads to equalities $ln(q) = c(l)$ for $E_R = \beta(l)$ and $c(l) = -(\alpha - 1)\,ln\left(\frac{E_R^0}{\beta(l)}\right)$ for $q = 1$, where E_R^0 is the energy dissipated with probability 1 (see below). The scaling factor $\beta(l)$ can be estimated subject to the constraint $(\alpha - 1)ln\left(\frac{\beta(l)}{E_R^0}\right) = -ln(N)$ or $ln(N) = (\alpha - 1)ln\left(\frac{E_R^0}{\beta(l)}\right)$ (3); then $c(l) = -ln(N)$. Thus, it can be assumed that $Nq = (E_R/\beta(l))^{\alpha-1}$ (4). Therefore, we can write Eq. 1 as $f\left(E_R \mid \beta(l), \alpha\right) = \frac{\alpha}{\beta(l)}\left(\frac{E_R}{\beta(l)}\right)^{\alpha-1} e^{-\left(\frac{E_R}{\beta(l)}\right)^{\alpha-1}} e^{-\frac{E_R}{\beta(l)}}$ or

$$f\left(E_R \mid \beta(l), \alpha\right) = \begin{cases} \frac{\alpha}{\beta(l)}\left(\frac{E_R}{\beta(l)}\right)^{\alpha-1} e^{-\left(\frac{E_R}{\beta(l)}\right)^{\alpha}} & E_R > 0 \\ 0 & E_R \leq 0 \end{cases} \qquad (5)$$

With cumulative probability distribution $F\left(E_R \mid \alpha, \beta\right) = 1 - e^{-\left(\frac{E_R}{\beta(l)}\right)^{\alpha}}$ (6). Since methylation changes can take place with random fluctuations in thermal noise, the scaling parameter $\beta(l)$ can be set equal to the average energy per DNA molecule in thermal equilibrium. That is, $\beta(l) = \varphi(l)k_BT$ (7), where $\varphi(l)$ expresses the contribution of all degrees of freedom to the average energy per molecule as a function of genomic region length l. Now, the physical meaning of energy E_R^0 derives after substitution of $\beta(l)$ given by Eq. 7 in Eq. 3. Explicitly, under the constraint expressed by Eq. 3, we have $E_R^0 = \sqrt[\alpha-1]{N}\beta(l) = \sqrt[\alpha-1]{N}\varphi(l)k_BT$, i.e., E_R^0 is the average energy per molecule contributed by all the degrees of freedom in N genomic regions of length l.

Derivation assuming a Poisson process

We assume the CDM changes induced by thermal fluctuation follow a Poisson process. Since Poisson is a limiting case of binomial process, the former inherits properties of

independence from the underlying binomial process. That is, given a Poisson process, the probability that the value of energy E_R can be dissipated exactly *n* times in *N* genomic regions is given by the binomial distribution: $B(n|N,q)=\frac{N!}{n!(N-n)!}q^n(1-q)^{N-n}$. The binomial distribution can be analyzed as a function of the expected number of times that energy $E_R \in \left[E_R^{i-1}, E_R^i\right)$ can be dissipated in *N* genomic regions $\nu = Nq$:

$B(n|N)=\frac{N!}{n!(N-n)!}\left(\frac{\nu}{N}\right)^n\left(1-\frac{\nu}{N}\right)^{N-n}$. Then, as the number of genomic regions becomes large enough, $B(n|N)$ approaches Poisson distribution $P(n|\nu)=\frac{\nu^n}{n!}e^{-\nu}$.

$$
\begin{aligned}
B_\nu(n) &= \lim_{N\to\infty} B(n|N) = \lim_{N\to\infty} \frac{N(N-1)\ldots(N-n+1)}{n!}\frac{\nu^n}{N^n}\left(1-\frac{\nu}{N}\right)^N\left(1-\frac{\nu}{N}\right)^{-n} \\
&= \lim_{N\to\infty} \frac{N(N-1)\ldots(N-n+1)}{N^n}\frac{\nu^n}{n!}\left(1-\frac{\nu}{N}\right)^N\left(1-\frac{\nu}{N}\right)^{-n} \\
&= 1\bullet\frac{\nu^n}{n!}e^{-\nu}\bullet 1 = \frac{\nu^n}{n!}e^{-\nu}
\end{aligned}
$$

For a large number of genomic regions, the probability that a particular value of energy E_R would be dissipated at least once in *N* genomic regions will be $P(1|\nu)=\nu e^{-\nu}$. It should then be expected that energies E_R with high probabilities $P(1|\nu)$ will be observed more frequently, i.e., the frequencies $f(E_R)$ are proportional to probabilities $P(1|\nu)$: $f(E_R)\propto Nq e^{-Nq}$. Then, after considering $f(E_R)\propto e^{-\left(\frac{E_R}{\beta(l)}\right)}$ (Figs. 1 & 2), we retrieve Eq.1: $f(E_R)=\frac{\alpha}{\beta(l)}Nq e^{-Nq}e^{-\left(\frac{E_R}{\beta(l)}\right)}$ and the rest of the reasoning to derive Eq. 5 follows as presented in the previous section.

Testing distribution of data

To expedite testing of the distribution of the I_R data, we have provided a homemade R script for a function called "fitCDF". This function requires previous installation of the R packages "minpack.lm" and "numDeriv". We provide two files of data to illustrate our analyses: 1) "*Four_ecotypes_CGs_IG_2000bp.RData*" and 2) "*Four_ecotypes_CGs_IG_5000bp.RData*", which contain GRanges objects (created with R package "GenomicRanges") with the partition of four Arabidopsis methylomes (four ecotypes) into non-overlapping windows of 2000 and 5000 bp, respectively. A small R script to visualize these data is given below.

```
library(GenomicRanges)
setwd( "~/yourworking directory/" )
source( " fitCDF.R" )
load( "Four_ecotypes_CGs_IG_5000bp.RData" )
> IG
GRanges object with 23683 ranges and 4 metadata columns:
                seqnames               ranges strand   |               Er_0               Fr_2
                   <Rle>            <IRanges>  <Rle>   |          <numeric>          <numeric>
         Chr1_1     Chr1       [    1,  5000]      *   |   8.80737483088559  -4.24430261119046
      Chr1_5001     Chr1       [ 5001, 10000]      *   |  0.490502525222088  -4.81099325075313
     Chr1_10001     Chr1       [10001, 15000]      *   |  -1.81127812445913  -1.40769688076302
     Chr1_15001     Chr1       [15001, 20000]      *   |   8.90901082556418   4.37532847136919
     Chr1_20001     Chr1       [20001, 25000]      *   |   11.7345678273271 0.0477567318121585
            ...      ...                  ...    ... ...                ...                ...
  Chr5_26955001     Chr5 [26955001, 26960000]      *   |  -8.55588937692415  -4.70259605385437
  Chr5_26960001     Chr5 [26960001, 26965000]      *   |   3.73610493864898    -2.671734377967
  Chr5_26965001     Chr5 [26965001, 26970000]      *   |   6.01465742862354   5.49410161054422
  Chr5_26970001     Chr5 [26970001, 26975000]      *   |   1.82337933117724   6.09546633554899
  Chr5_26975001     Chr5 [26975001, 26980000]      *   |  0.382428446508557   7.10709855580481
                          Gifu_2          Seattle_0
                       <numeric>          <numeric>
         Chr1_1  -5.13192978402208  -4.06900380786532
      Chr1_5001  -13.9267152828099 -0.310614868676782
     Chr1_10001 -0.453808602174457  0.223212946461473
     Chr1_15001  -1.22003859611185   5.81747772270944
     Chr1_20001 -0.824852003187666   11.7143862528797
            ...                ...                ...
  Chr5_26955001  -10.4271281185123 -0.528917490574309
  Chr5_26960001  -2.06264096524125  0.998900086746565
  Chr5_26965001  -8.26703876113279    11.379301729585
```

The variable carried by the GRanges object is called "IG". Herein, we'll write an example with the Arabidopsis ecotype "Seattle_0".

```
dG = mcols( IG )
ig = abs( dG[,"Seattle_0"] ) # select  data from "Seattle_0"
fit = fitCDF( ig, plot.num = 2 ) # It will yield the plot of the
first two best distributions.
```

Running this piece of script will yield:

```
> fit <- fitCDF( ig, plot.num = 2 )
Loading required package: minpack.lm
Loading required package: numDeriv
Fitting Normal distribution...Done.
Fitting Log-normal distribution...Done.
Fitting Generalized Normal distribution...Done.
Fitting Laplace distribution...Done.
Fitting Gamma distribution...Done.
Fitting 3P Gamma distribution...Done.
Fitting Generalized Gamma distribution...Done.
Fitting Weibull distribution...Done.
Fitting 3P Weibull distribution...Done.
Fitting Beta distribution...Done.
Fitting 3P Beta distribution...Done.
Fitting 4P Beta distribution...Done.
Fitting Generalized Beta distribution...Done.
Fitting Rayleigh distribution...Done.
Fitting Exponential distribution...Done.
Fitting 2P Exponential distribution...Done.
 * Estimating Studentized residuals for distribution #  1
 * Plot #  1 ...
 * Estimating Studentized residuals for distribution #  2
 * Plot #  2 ...
** Done ***
```

Depending on machine computational power, the fit of Generalized Gamma (GG) distribution will vary in duration. Following return of plots, a list object with the following values is provided:

- aic: Akaike information criterion
- fit: list of results of fitted distribution, with parameter values
- bestfit: the best fit distribution according to AIC
- fitted: fitted values from the best fit
- rstudent: studentized residuals
- residuals: residuals

The first plot corresponds to the best model according to Akaike information criterion (AIC), which in the current case is GG distribution (Fig. 5). The main problem with the fitting is that the scale parameter does not make physical sense, since it approaches zero. From the experimental data shown in Figs 1 to 2, we know that the scale parameter differs from zero and increases with size of the genomic region. Thus, although the AIC indicates that this is the best model, it is discarded from a physical standpoint. This outcome is an artifact of the numerical fitting algorithm. The second best model is the 3-parameters Weibull distribution (Fig. 6).

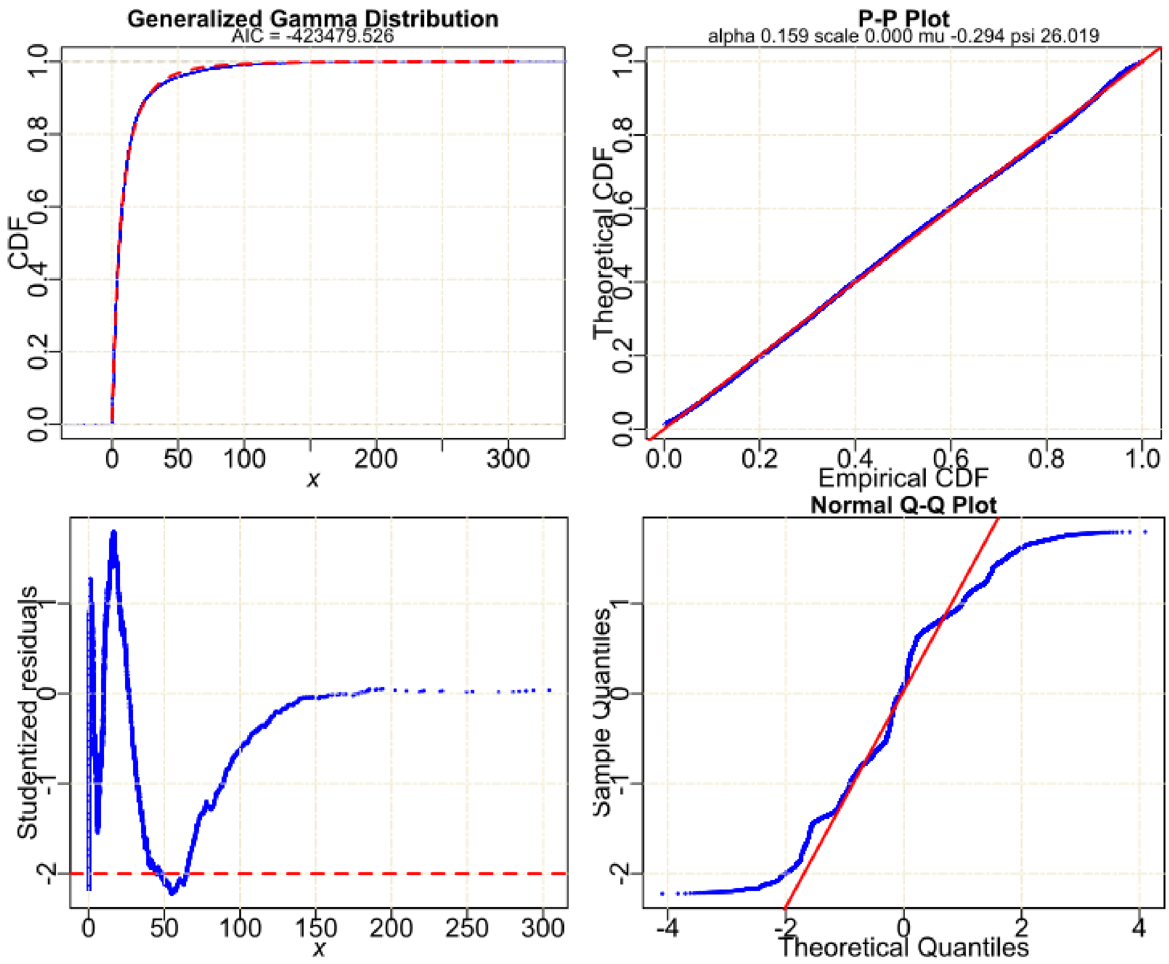

Figure 5. The best fit model according to AIC for I_R data from "Seattle_0". This model is discarded since the scale parameter is very close to zero.

To get the list of all fitted results, we can type: fit$fit. For the fitting result of the 3-parameter Weibull distribution we can type:

```
> fit$fit$"3P Weibull"
Nonlinear regression via the Levenberg-Marquardt algorithm
parameter estimates: 0.193733766873807, 0.803545758745314,
8.65454571509012
residual sum-of-squares: 0.0007789
reason terminated: Relative error in the sum of squares is at most
`ftol'
```

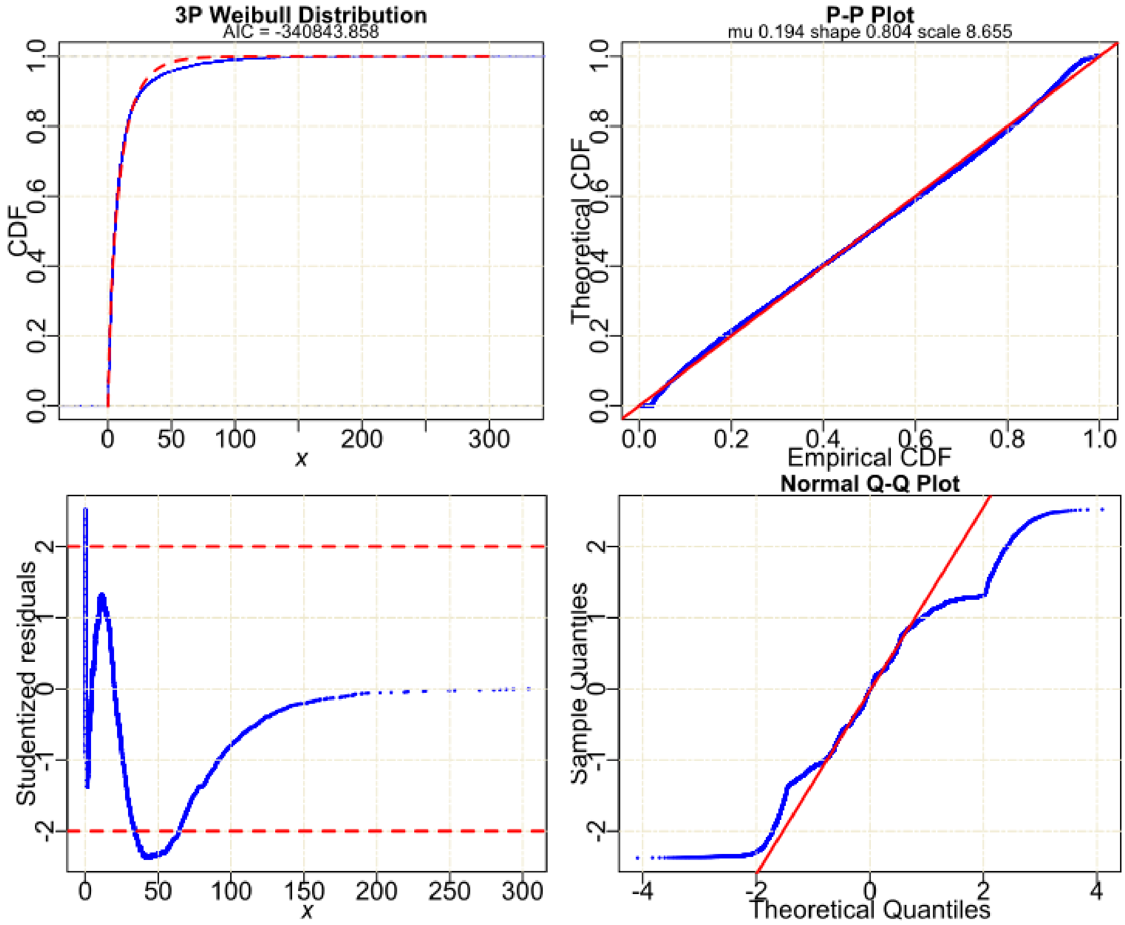

Figure 6. The second best fit model according to AIC for I_R data from "Seattle_0".

The model summary is obtained by typing:

```
> summary(fit$fit$"3P Weibull")

Parameters:
       Estimate   Std. Error t value   Pr(>|t|)
mu     0.1937338  0.0009188  210.9     <2e-16 ***
shape  0.8035458  0.0003436  2338.7    <2e-16 ***
scale  8.6545457  0.0029626  2921.3    <2e-16 ***
---
Signif. codes:  0 '***' 0.001 '**' 0.01 '*' 0.05 '.' 0.1 ' ' 1

Residual standard error: 0.0001814 on 23680 degrees of freedom
Number of iterations to termination: 11
Reason for termination: Relative error in the sum of squares is at most
`ftol'.
```

About Q-Q norm and Kolmogorov-Smirnov goodness of fit problems for large data sets

In Figs. 5 to 6, it appears that the expected normal distribution for the Studentized residuals derived from the non-linear fit of I_R has some problems. However, the problem is neither in the experimental data nor in the non-linear fit, but in the Q-Q norm plot when the size of the dataset is large enough. An analogous issue is found for Kolmogorov-Smirnov goodness of fit.

We supplied an R function "`qq.weibull`" to illustrate the issue by simulation. This function generates random numbers according to a specified Weibull distribution and then a small white-noise is added by using the R-base function "`jitter`" (see the details of this function in R by typing `?jitter`).

- ✓ **Example 1**:

```
source("qq.weibull.R")
# Example 1
i = 38 # To set a random seed

# A simulation based on 10^4 random empirical values with Weibull
#distribution with parameters:
# alpha = 0.6831651, scale = 2.5114992, mu = 0.01

qq.weibull( s = 1, m = 4, seed = i )
```

This piece of script will yield Fig. 7 and the tables below

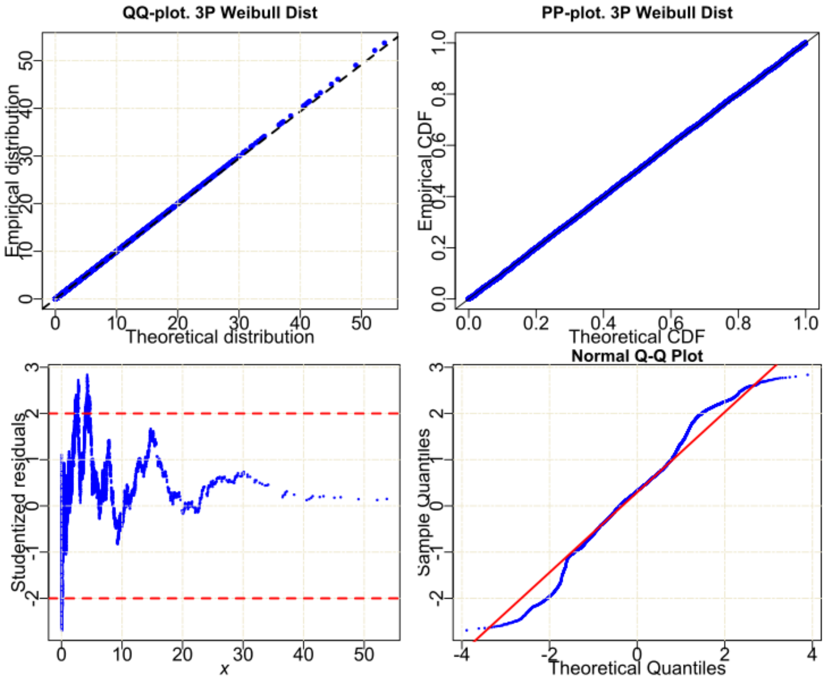

Figure 7. Simulation to illustrate the effect of the data size on QQ-norm plot. Sample size: 10000.

```
> qq.weibull( s = 1, m = 4, seed = i )
$lm

Call:
lm(formula = p.teo ~ p.emp)

Residuals:
       Min         1Q     Median         3Q        Max
-0.0040990 -0.0010132  0.0000292  0.0010257  0.0042891

Coefficients:
             Estimate Std. Error  t value Pr(>|t|)
(Intercept) 1.437e-03  3.176e-05    45.25   <2e-16 ***
p.emp       9.958e-01  5.501e-05 18101.17   <2e-16 ***
---
Signif. codes:  0 '***' 0.001 '**' 0.01 '*' 0.05 '.' 0.1 ' ' 1

Residual standard error: 0.001588 on 9998 degrees of freedom
Multiple R-squared:      1,	Adjusted R-squared:      1 
F-statistic: 3.277e+08 on 1 and 9998 DF,  p-value: < 2.2e-16


$ks

	One-sample Kolmogorov-Smirnov test

data:  x.emp
D = 0.0057, p-value = 0.905
alternative hypothesis: two-sided
```

The QQ and PP plots, as well as the linear regression analysis “theoretical probabilities” versus “empirical probabilities” and Kolmogorov-Smirnov test, tell us that this fit is okay. However, we can see that the QQ-norm plot is reflecting a small issue (Fig. 7).

- ✓ **Example 2**:

The increment of the data size, retaining the same parameter setting, eliminates the problem:

```
# A simulation based on 2.3 * 10^4 random empirical values with Weibull
# distribution
qq.weibull( s = 2.3, m = 4, seed = i )
```

This sample size is consistent with the partition of Arabidopsis methylome into non-overlapping windows of 5000 bp (see the GRanges object above). Results are presented in Fig. 8. Now, QQ-norm plot reflects a real issue. This issue is also quantitatively shown by the Kolmogorov-Smirnov test, which rejects the normality hypothesis of the Studentized residuals (see below).

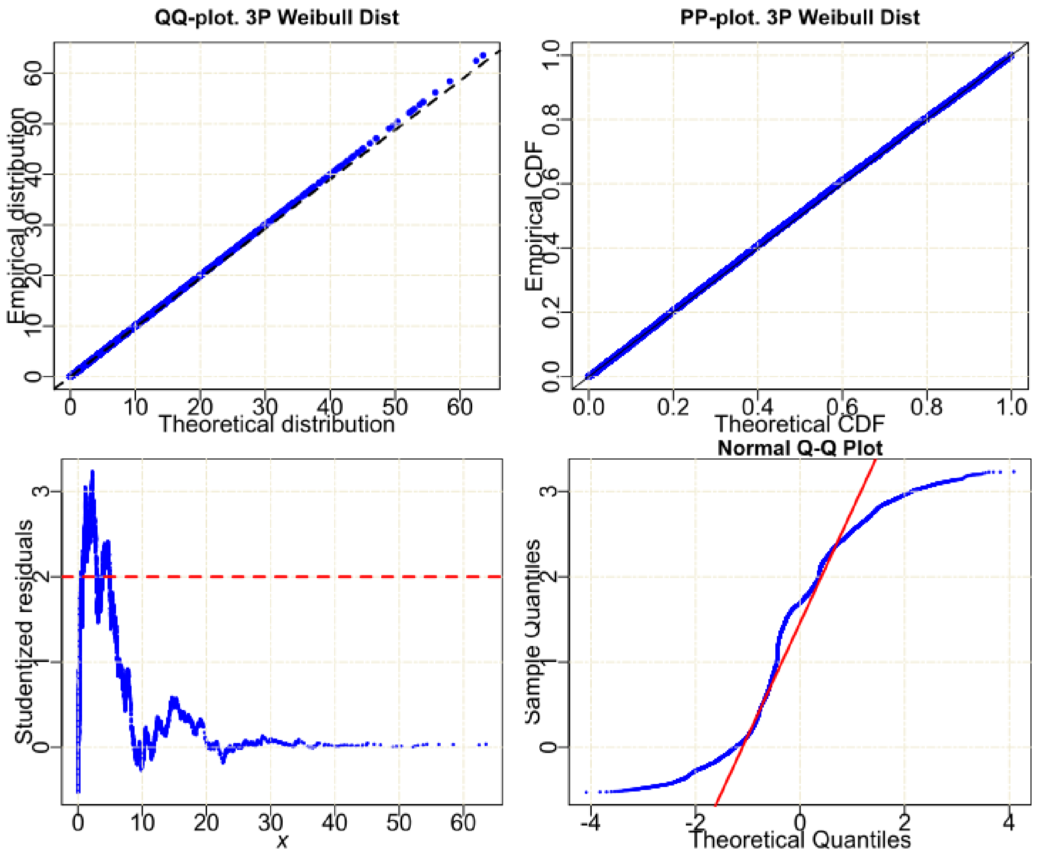

Figure 8. Simulation to illustrate the effect of the data size on QQ-norm plot. Sample size: 23000.

```
> qq.weibull( s = 2.3, m = 4, seed = i )
$lm

Call:
lm(formula = p.teo ~ p.emp)

Residuals:
       Min         1Q     Median         3Q        Max 
-0.0051559 -0.0026236 -0.0007266  0.0027158  0.0061380 

Coefficients:
              Estimate Std. Error  t value Pr(>|t|)    
(Intercept) -3.783e-03  4.011e-05   -94.33   <2e-16 ***
p.emp        9.984e-01  6.946e-05 14372.14   <2e-16 ***
---
Signif. codes:  0 '***' 0.001 '**' 0.01 '*' 0.05 '.' 0.1 ' ' 1 

Residual standard error: 0.003041 on 22998 degrees of freedom
Multiple R-squared:  0.9999,     Adjusted R-squared:  0.9999 
F-statistic: 2.066e+08 on 1 and 22998 DF,  p-value: < 2.2e-16


$ks

        One-sample Kolmogorov-Smirnov test

data:  x.emp 
D = 0.0099, p-value = 0.02118
alternative hypothesis: two-sided
```

This problem can be solved by applying a permutation test as described in Alastair Sanderson's web page: "Using R to analyse data: statistical and numerical data analysis with R" (http://www.sr.bham.ac.uk/~ajrs/R/r-analyse_data.html).

```
> qq.weibull( s = 2.3, m = 4, plot = FALSE, num.permt = 999, seed = i )
*** Performing permutation test for KS statistic.  999  permutations...
$lm
Call:
lm(formula = p.teo ~ p.emp)
Residuals:
       Min         1Q     Median         3Q        Max 
-0.0051559 -0.0026236 -0.0007266  0.0027158  0.0061380 

Coefficients:
              Estimate Std. Error  t value Pr(>|t|)    
(Intercept) -3.783e-03  4.011e-05   -94.33   <2e-16 ***
p.emp        9.984e-01  6.946e-05 14372.14   <2e-16 ***
---
Signif. codes:  0 '***' 0.001 '**' 0.01 '*' 0.05 '.' 0.1 ' ' 1 

Residual standard error: 0.003041 on 22998 degrees of freedom
Multiple R-squared:  0.9999,     Adjusted R-squared:  0.9999 
F-statistic: 2.066e+08 on 1 and 22998 DF,  p-value: < 2.2e-16

$ks
        One-sample Kolmogorov-Smirnov test
data:  x.emp 
D = 0.0099, p-value = 0.02118
alternative hypothesis: two-sided

$Permutation.p.value
p-value 
  1 
```

The p-value obtained does not reject the normality hypothesis of the Studentized residuals.
