# Supplementary figures and images for: Information Thermodynamics of Cytosine DNA Methylation
Source: PLoS One. 2016 Mar 10;11(3):e0150427. doi: 10.1371/journal.pone.0150427 (PMC4786201; doi:10.1371/journal.pone.0150427)

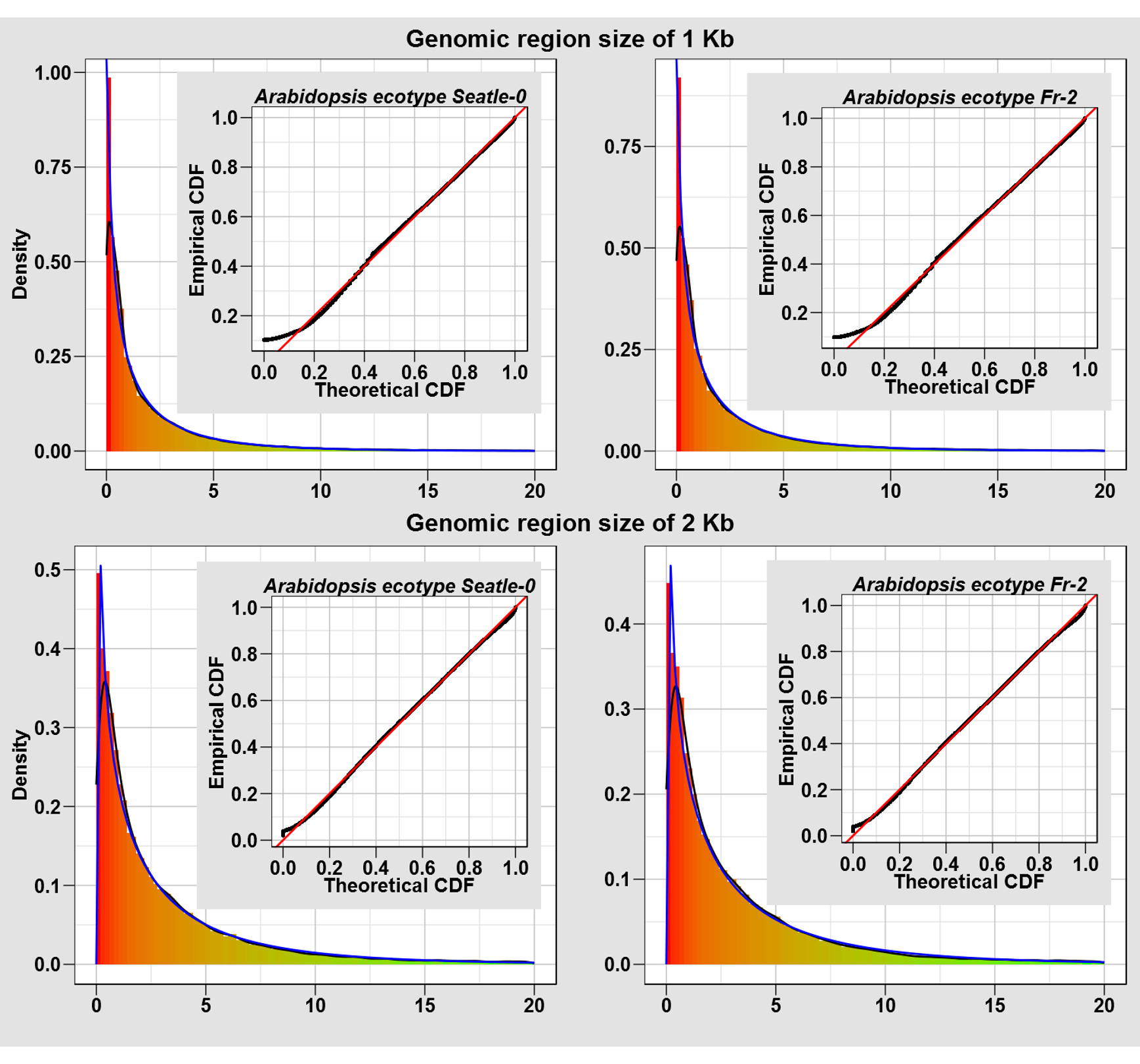

Supplement: S1 Fig — The empirical CDF of IR departs from the theoretical CDF given in Eq 14 for genomic region sizes l below 2 Kb. The analysis of the PDFs and the CDFs reveals a significant increase in the frequency of genomic regions with very small information changes (IR values close to zero) as the methylome is split into regions with sizes l < 2 Kb. PDF curves corresponding to the theoretical parameters estimated from Eq 14 (blue), and kernel density estimations (e.g.,“empirical” estimations that depend on the algorithm, kernel and bandwidth used) are also shown (black). (TIF) [file pone.0150427.s008.tif]

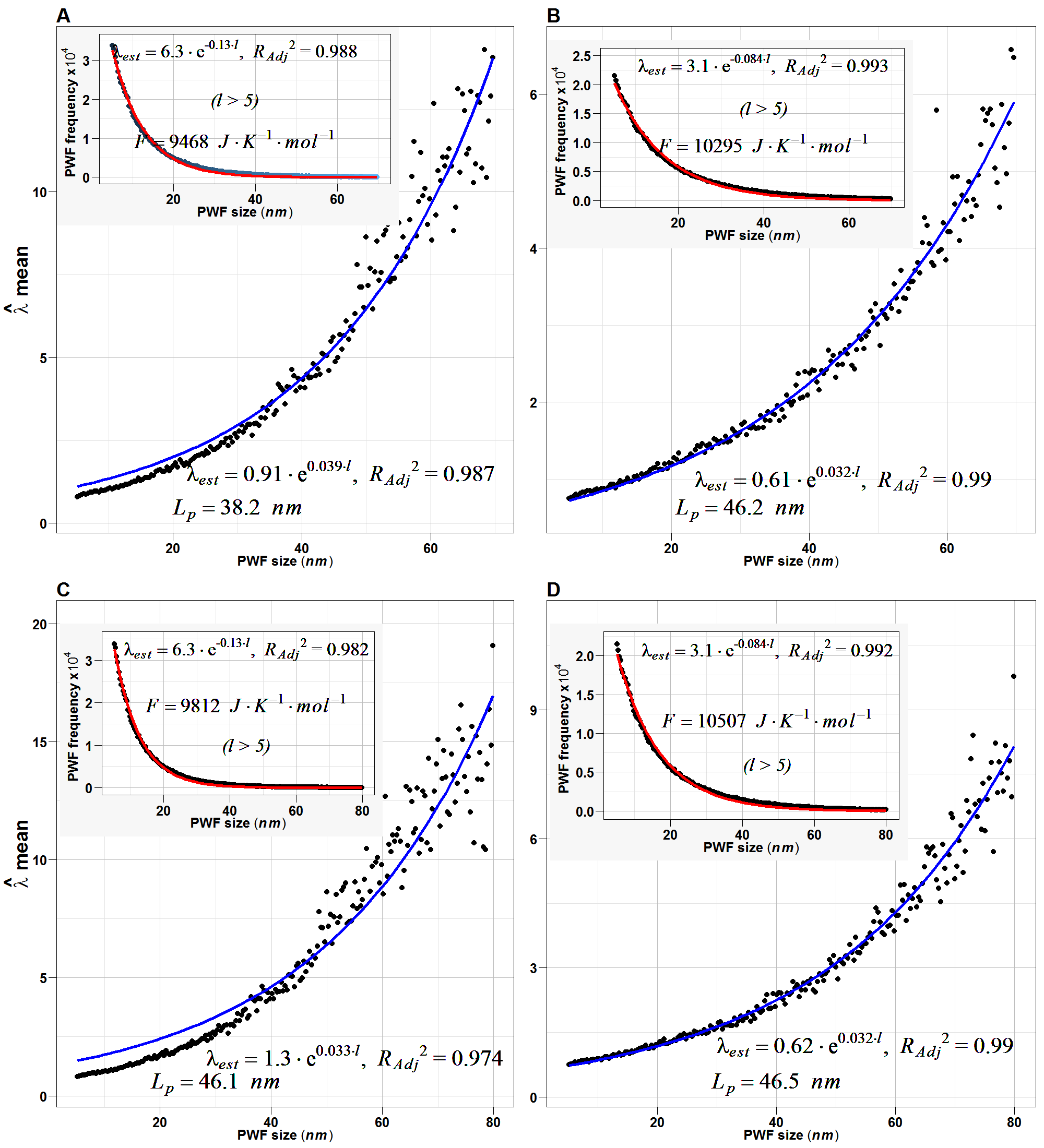

Supplement: S2 Fig — Statistical trends were estimated in 150 Arabidopsis ecotypes [41] considering all CDM contexts. (A) and (B), exponential region of the relationship λ^¯(l) vs l in the range of PWF from 5 to 70 nm for the partitions S6 and S7, respectively. (C) and (D), exponential regions of λ^¯(l) vs l in the range of PWF from 5 to 80 nm for the partitions S6 and S7, respectively. The exponential behavior is consistent with Eq 22 (23), which permits the estimation of the DNA persistence length Lp by means of Eq 24. The exponential decay law predicted by Eq 33 was verified (subplots mean of PWF-frequency (f) vs l in panels A to D). The estimated value of the Helmholtz free energy ΔF = RT ln Z(γ) (Eq 34) at 298.15 K of temperature is indicated. (TIF) [file pone.0150427.s009.tif]
